# Differing Responses to *Phytophthora cinnamomi* Infection in Susceptible and Partially Resistant *Persea americana* (Mill.) Rootstocks: A Case for the Role of Receptor-Like Kinases and Apoplastic Proteases

**DOI:** 10.3389/fpls.2022.928176

**Published:** 2022-06-28

**Authors:** Robert Backer, Juanita Engelbrecht, Noëlani van den Berg

**Affiliations:** ^1^Hans Merensky Chair in Avocado Research, University of Pretoria, Pretoria, South Africa; ^2^Department of Biochemistry, Genetics and Microbiology, Faculty of Natural and Agricultural Sciences, University of Pretoria, Pretoria, South Africa; ^3^Forestry and Agricultural Biotechnology Institute, Faculty of Natural and Agricultural Sciences, University of Pretoria, Pretoria, South Africa

**Keywords:** dual RNA-sequencing, *Phytophthora cinnamomi*, avocado, receptor-like kinase, protease, time-course

## Abstract

The hemibiotrophic plant pathogen *Phytophthora cinnamomi* Rands is the most devastating pathogen of avocado (*Persea americana* Mill.) and, as such, causes significant annual losses in the industry. Although the molecular basis of *P. cinnamomi* resistance in avocado and *P. cinnamomi* virulence determinants have been the subject of recent research, none have yet attempted to compare the transcriptomic responses of both pathogen and host during their interaction. In the current study, the transcriptomes of both avocado and *P. cinnamomi* were explored by dual RNA sequencing. The basis for partial resistance was sought by the inclusion of both susceptible (R0.12) and partially resistant (Dusa®) rootstocks sampled at early (6, 12 and 24 hours post-inoculation, hpi) and late time-points (120 hpi). Substantial differences were noted in the number of differentially expressed genes found in Dusa® and R0.12, specifically at 12 and 24 hpi. Here, the partially resistant rootstock perpetuated defense responses initiated at 6 hpi, while the susceptible rootstock abruptly reversed course. Instead, gene ontology enrichment confirmed that R0.12 activated pathways related to growth and development, essentially rendering its response at 12 and 24 hpi no different from that of the mock-inoculated controls. As expected, several classes of *P. cinnamomi* effector genes were differentially expressed in both Dusa® and R0.12. However, their expression differed between rootstocks, indicating that *P. cinnamomi* might alter the expression of its effector arsenal based on the rootstock. Based on some of the observed differences, several *P. cinnamomi* effectors were highlighted as potential candidates for further research. Similarly, the receptor-like kinase (RLK) and apoplastic protease coding genes in avocado were investigated, focusing on their potential role in differing rootstock responses. This study suggests that the basis of partial resistance in Dusa® is predicated on its ability to respond appropriately during the early stages following *P. cinnamomi* inoculation, and that important components of the first line of inducible defense, apoplastic proteases and RLKs, are likely to be important to the observed outcome.

## Introduction

Avocado (*Persea americana* Mill.) is an economically important fruit crop belonging to Lauraceae. This family is arguably the most economically important family in Laurales, consisting of over 2,500 species with a wide tropical and subtropical distribution (Kostermans, 1957; Gentry, 1988; Li, 1995; van der Werff and Richter, 1996; Chanderbali et al., 2001; Song et al., 2016). Of these, avocado contributes significantly to the global economy with production surpassing 7 million tons in 2019 and an estimated gross production value of $6.56 billion (constant 2014–2016, int. $; http://www.fao.org/faostat/en/#home). In recent years, avocados have gained popularity because of their nutritional profile coupled with numerous health benefits associated with their consumption (Hurtado-Fernández et al., 2018). However, the production of avocado is severely threatened by the persistence of several pathogens in major avocado-growing regions of the world (van den Berg et al., 2021).

One of the most significant threats to avocado production is Phytophthora root rot (PhRR), caused by the oomycete *Phytophthora cinnamomi* Rands, a plant pathogen of global significance due to its effects on wild and cultivated plants, which presents a serious threat to the diversity and structure of natural ecosystems(Hardham and Blackman, 2018). *P. cinnamomi* is a hemibiotrophic oomycete, implying that its interaction with the host is initially biotrophic, following host colonization, after which a necrotrophic phase is invoked. In avocado, *P. cinnamomi* invades the sub-surface feeder roots where, in susceptible rootstocks, the root structure is rapidly colonized and functionally damaged, limiting water and nutrient uptake. Control and management of PhRR spread remain a challenging endeavor because of *P. cinnamomi's* broad host range coupled with the ability of tolerant or partially resistant plants to remain asymptomatic. Chemical control of *P.cinnamomi* has predominantly been conducted using phosphites in the form of sprays or trunk injections (Pegg et al., 1985; Hardy et al., 2001). However, decreased sensitivity to treatment with phosphites and the questionable sustainability of clearing infested orchards point to integrated approaches as the only viable method for PhRR control and management (Dobrowolski et al., 2008; Wolstenholme and Sheard, 2010). These integrated approaches include methods such as use of tolerant or partially resistant rootstocks such as Dusa®, together with chemical control and efficient orchard management (Hardham, 2005; Wolstenholme and Sheard, 2010). Nonetheless, the impact of PhRR remains because of reduced fruit size, quality and shelf life, decreasing yield and, in the case of severe infection, killing of trees (Dann et al., 2013).

To augment the use of partially resistant rootstocks as a control measure for future management of PhRR, a comprehensive understanding of the *P. americana-P. cinnamomi* interaction is essential (van den Berg et al., 2021). One way of understanding host-pathogen interactions is quantifying gene expression in both the pathogen and the host during infection. This would invariably lead to a better understanding of *P. cinnamomi* molecular responses during infection, paving the way for the development of novel targets for precise and viable control measures. Meanwhile, understanding host responses could aid in the development or selection of novel PhRR-resistant rootstocks.

The availability of high-quality omics data to study the *P. americana*-*P. cinnamomi* pathosystem has improved significantly over the past few years. The newest *P. cinnamomi* genome represents a significant improvement over previous genome assemblies (Engelbrecht et al., 2021). Similarly, the *P. americana* West-Indian pure accession (WI) rootstock genome from the Avocado Genome Consortium boasts a chromosome-level reference assembly (Avocado Genome Consortium, article in preparation). These resources are invaluable for future studies on the *P. americana*-*P. cinnamomi* interaction at the genetic level. There are, however, only a few resources available for understanding the expression of genes for this pathosystem on a global scale (Mahomed and van den Berg, 2011; Reeksting et al., 2014, 2016; Reitmann et al., 2017; van den Berg et al., 2018b; Engelbrecht et al., 2021). Notably, insights into this pathosystem could be significantly improved by high-throughput transcriptome sequencing such as RNA-seq. This technology profiles transcriptomes using deep-sequencing technologies, enabling quantification of gene expression and providing a novel understanding of changing RNA expression levels that occur in response to external stimuli (Martin et al., 2013). In particular, dual RNA sequencing data, which allow for investigations on the interaction of both host and pathogen during the process of infection (Westermann et al., 2012; Wolf et al., 2018), could further aid in understanding this important pathosystem.

To understand any pathosystem, one needs to only consider the evolutionary arms race that exists between a pathogen and a host. Generally, plants recognize pathogens through one of two methods, the first is by recognition of pathogen-associated molecular patterns (PAMPs; Davis and Hahlbrock, 1987; Zipfel and Felix, 2005). These molecular patterns are recognized by membrane-bound pattern recognition receptors (PRRs), leading to activation of the innate immune-response, pattern-triggered immunity (PTI). Notably, most PRRs are either transmembrane receptor-like kinases (RLKs) or receptor-like proteins (RLPs) and, as such, constitute an essential part of the pathogen-sensing machinery in plants (Böhm et al., 2014; Couto and Zipfel, 2016; Yu et al., 2017). Here, plant proteases have important roles in the signaling process, both prior to and following the recognition of PAMPs or host-derived damage-associated molecular patterns (DAMPs; Hou et al., 2018; Godson and van der Hoorn, 2021). Intriguingly, pathogens have been known to evade recognition by PRRs through the actions of effector proteins, some of which seem to trick the host plant into activating RLKs, which are involved in growth and development (Dou and Zhou, 2012). In turn, pathogen effectors are often recognized either directly or indirectly by various resistance (R) proteins, culminating in the second stronger inducible immune-response, effector-triggered immunity (ETI; Jones and Dangl, 2006; Monteiro and Nishimura, 2018). Typically, ETI leads to a specialized form of localized programmed cell death (PCD), known as the hypersensitive response (HR), to arrest pathogen spread (Cui et al., 2015). As such, ETI is known to be effective against biotrophic or hemibiotrophic pathogens but not against necrotrophs (Glazebrook, 2005). In plants, the largest group of R proteins are that of cytoplasmic nucleotide-binding leucine-rich repeat (NLR) proteins (McDowell and Woffenden, 2003). Notably, the full complement of avocado *NLR*s was recently described using some of the RNA-seq data presented in this study (Fick et al., 2022).

To gain a better understanding of the *P. americana*- *P. cinnamomi* interaction at a molecular level, a comprehensive root transcriptomic analysis was performed using dual RNA sequencing technology. The experimental approach included the use of both a susceptible (R0.12) and a partially resistant avocado rootstock (Dusa®), inoculated with *P. cinnamomi* and harvested at 6, 12, 24, and 120 h post-inoculation (hpi). This approach allows for the simultaneous investigation of molecular components that govern susceptibility and/or resistance in avocado roots over time, as well as pathogen virulence strategies, at both the biotrophic and necrotrophic life stages and the transition between the two. We utilized the reference genomes for *P. americana* WI pure accession and *P. cinnamomi* GKB4 for RNA-seq read alignment. Differentially expressed genes (DEGs) were identified, and the investigation implicated several *P. americana* genes encoding for pathogenesis-related (PR) genes, receptor-like kinases (RLKs) and proteases, and others in the defense response against *P. cinnamomi*. Correspondingly, several *P. cinnamomi* effector coding genes were identified and linked to the infection process including but not limited to RxLRs, elicitins, extra-cellular protease inhibitors (EPIs), and xyloglucan-specific endo-beta-1,4-glucanases (XEGs). Most importantly, the analyses conducted in this study showed that Dusa® exhibited drastic responsive gene expression differences during the biotrophic-necrotrophic transition phase of infection when compared to the susceptible host. This observation suggests that a sweeping responsive change in gene expression is the molecular basis of partial resistance.

This is the first study that conducted dual RNA-seq to examine the global gene expression of both *P. cinnamomi* and two inoculated avocado rootstocks of varying susceptibility during infection. The study provides the most comprehensive overview of avocado defense response pathways to date and a glimpse at the arsenal of virulence factors utilized by *P. cinnamomi* to promote disease. Besides candidates for functional validation, the results generated by this study will provide resources for future identification of markers for improved rootstock breeding and screening.

## Materials and Methods

### Plant and Pathogen Material

Both *in vitro* and *in planta* materials were used for this research. For the *in vitro* material, *P. cinnamomi* (isolate GBK4) was grown on 5% V8 agar for 5 days at 25°C in the dark. Mycelia were harvested and snap-frozen in liquid nitrogen before being stored at −80°C until RNA extraction. For the *in planta* material, clonal avocado plantlets approximately 2 years old were obtained from Westfalia Technological Services (Tzaneen, Limpopo, South Africa). A zoospore suspension of *P. cinnamomi* (isolate GKB4) was produced as described in Engelbrecht and van den Berg (2013). Both partially resistant (Dusa®) and susceptible (R0.12) avocado rootstocks were inoculated using the zoospore suspension (1.4 x 10^5^ zoospores/ml) or dH_2_0 (mock-inoculation), submerging the roots and lower stem for 2 h. Following inoculation, all the plantlets were re-planted in 1.5-L plastic bags containing a 1:1 perlite:vermiculite mixture. Root samples were harvested for three replicate samples, with three plantlets per replicate at each time-point: 6, 12, 24, and 120 hpi. Due to resource limitations, control plants were only harvested at 24 hpi. The harvested materials were snap-frozen in liquid nitrogen before being stored at −80°C until RNA extraction.

### RNA Extraction and Sequencing

All the sample materials were processed to a fine powder using the IKA®Tube Mill control(IKA®, Staufen, Germany).A modified CTAB RNA extraction protocol was used to extract total RNA from all the samples as described in Engelbrecht and van den Berg (2013). RNA concentration was quantified using a Nanodrop ND−1000 spectrophotometer (Nanodrop Technologies Inc., Montchanin, DE, United States). RNA integrity was assessed on a 2% TAE agarose gel under non−denaturing conditions. Potential contaminating DNA was removed from total RNA using RNase−free DNase (Fermentas Life Sciences, Hanover, MD, United States) according to the manufacturer′s guidelines. DNAse−treated RNA was then purified using an RNeasy®Mini−Elute® cleanup kit (Qiagen, Valencia, CA, United States) according to the manufacturer′s guidelines. Purified total RNA quality was assessed on Agilent 2100 Bioanalyzer (Agilent Technologies, CA, United States). Finally, 1 μg purified RNA from each biological replicate was combined to constitute the samples used for each of the respective libraries (Supplementary Table 1). Purified RNA was shipped to Macrogen (Macrogen Inc., Seoul, GG, South Korea) for sequencing on an Illumina HiSeq platform using the PE150 mode with a minimum raw read count of 80 million per sample.

### RNA Sequencing Quality Control and Genome Alignment

Raw RNA-seq data were filtered using Trimmomatic v0.39 [trimming parameters: ILLUMINACLIP: TruSeq3-PE.fa:2:30:10:2:true LEADING:3 TRAILING:3 SLIDINGWINDOW:4:15 MINLEN:36] to remove low-quality bases and Illumina sequencing adapters (Bolger et al., 2014). The read quality before and after trimming was assessed using FastQC v0.11.9 (Andrews, 2010) and summarized using MultiQC v1.9 (Ewels et al., 2016). The *P. americana* WI pure accession genome was obtained from the Avocado Genome Consortium (Avocado Genome Consortium, article in preparation). The *P. cinnamomi* GKB4 genome was obtained from Engelbrecht et al. (2021). The genomes were concatenated and used for RNA-seq data alignment to eliminate alignment bias from conserved sequences. Trimmed RNA-seq reads were aligned to the combined *P. americana-P.cinnamomi* genome using HISAT2 v2.0.6 (Kim et al., 2019). The resultant alignment files were processed and combined using samtools (Li et al., 2009) and used to obtain high-confidence splice junctions from Portcullis v1.2.0 (Mapleson et al., 2018). A second HISAT2 alignment was performed using the high confidence splice junctions obtained from Portcullis. Alignment statistics were calculated using samtools idxstats and flagstat. Transcripts per million (TPMs) were calculated using StringTie v2.1.4 (Pertea et al., 2015).

### Differential Gene Expression Analyses

Read counts were quantified at the gene level using featureCounts v2.0.1 (Liao et al., 2014), informed by the genome annotation file of either *P. americana* or *P. cinnamomi* in order to separate the reads from each of the respective genomes. Read data were imported into R 4.0.4 (R Core Team, 2021) and analyzed using DeSeq2 v1.32.0 (Love et al., 2014). Data for transcripts in each library that fell below 10 reads were removed, and transcripts without any read data were removed from the data set. Mock-inoculated *P. americana* libraries for R0.12 or Dusa® were set as reference libraries for the *P. americana* gene sets against the inoculated *P. americana* R0.12 or Dusa® rootstock libraries, respectively. The mycelial libraries were set as the reference for the *P. cinnamomi* gene sets against the inoculated *P. americana* rootstock libraries. DEGs were identified by Wald test, and multiple hypotheses testing correction was conducted using the Benjamini-Hochberg (BH) false discovery rate (FDR) method. Significantly DEGs were defined using an FDR cutoff (adjusted *p*-value) of <0.05 and a log_2_(fold change) of more than 1 (upregulated) and < −1 (downregulated). The *varianceStabilizingTransformation* (VST) function in the DESeq2 package was used to transform the DESeq2 normalized data. The VST data were used for principal component analyses (PCAs) of the top 500 variable genes using *plotPCA*. Hierarchical clustering of 50 genes with the highest variation across the samples was conducted using *hclust* and dendextend (Galili, 2015), followed by visualization using *heatmap.2* in gplots v3.1.1 (Warnes et al., 2009). Additionally, a list of the top 100 variable genes from the *P. americana* dataset was exported for further analyses.

Additional time course analyses were also conducted for both *P. americana* and *P. cinnamomi* using TCseq v1.16.0 (Wu and Gu, 2021) for downstream temporal pattern analyses. Raw count data (featureCounts) for both *P. americana* and *P. cinnamomi* were imported into R separately and divided into two datasets, one for each rootstock. Differential analyses were conducted using the *DBanalysis* function in TCseq, filtering out sample data points with <10 reads and any genes in which less than two samples contain read counts. A time course table was generated using Reads Per Kilobase Million (RPKM) normalized read counts. Significantly DEGs were defined using an FDR cutoff of <0.05 and a log_2_(fold change) of more than 1 and < −1. The Z-scores of DEGs were calculated and used for clustering analyses using fuzzy c-means (Futschik and Carlisle, 2005) with k = 5, as implemented by the *timeclust* function in TCseq. Clusters were visualized using the *timeclustplot* function.

### Gene Ontology Enrichment Analyses

*P. americana* and *P. cinnamomi* significant DEG lists from DESeq2 were imported into R 4.0.4 and split into up- and downregulated sets. Gene ontology enrichment was performed using GoSeq v1.44.0 (Young et al., 2010). Over- and under-representation of GO terms among DEGs in each gene set were calculated using the Wallenius approximation, and overrepresented GO terms with *p*-values <0.05 (BH FDR) were selected to constitute enriched GO term lists. Gene lists from each cluster obtained following TCseq clustering analyses were also subjected to GO enrichment using the same parameters. Lists of GO terms were reduced using REVIGO (Supek et al., 2011) and visualized in Tableau v2021.2.1.

### Significant Pathway, Gene Family, and Virulence Factor Identification

Predicted proteins from the *P. americana* WI pure accession genome were assigned to MapMan bins using both Mercator v3.6 and Mercator v4.0 applying default parameters and all available datasets (Lohse et al., 2014; Schwacke et al., 2019). Significant DEGs consequent to DeSeq2 analyses, from each time point and rootstock, were input into MapMan v3.6.0RC1 (Schwacke et al., 2019) to visualize and compare pathways and gene families for which expression differs in the rootstocks over time and between the rootstocks at defined time points following inoculation with *P. cinnamomi*. Alternatively, DEGs were visualized using heatmaps generated using pheatmap v1.0.12 (Kolde, 2012) in RStudio v1.4.1106 (RStudio Team, 2021). Localization prediction for predicted proteins of interest was performed using a lightweight deep neural network web service (Stärk et al., 2021). In order to identify potential *P. cinnamomi* virulence factors, predicted protein sequences from the corresponding *P. cinnamomi* TCseq cluster gene lists were compared to the Pathogen-Host Interactions (PHI) database v4.12 (Urban et al., 2019). This comparison was conducted using *blastp* in the *BLAST*+ suite (Camacho et al., 2009), with an Expect value (E) cutoff of 1.0e^−5^ and returning only the top match for each query.

## Results

### Transcript Expression Analyses

Following quality control, a total of 4.99 billion RNA sequencing reads were generated, with approximately 2.54 billion from the Dusa® libraries, 1.98 billion from the R0.12 libraries, and 475 million from the *P. cinnamomi* mycelial libraries (Supplementary Table 2). Thus, an average of 150 million filtered reads were obtained per library. Overall, 81.11% of the reads in the Dusa® libraries and 74.29% of the reads in the R0.12 libraries aligned to the *P. americana* genome. The read percentage from both Dusa® and R0.12 inoculated libraries that aligned to the *P. americana* genome was substantially lower at 120 h hpi in comparison to the earlier time points, 62.32% vs. 81.54%, coinciding with a sharp rise in the average read percentage aligning to the *P. cinnamomi* genome at 120 h hpi, 3.07% vs. 0.16% at earlier time points. An average of 0.89% of the reads in the inoculated sample libraries and 94.36% in the mycelial libraries aligned to the *P. cinnamomi* genome. On average, 0.004% of the reads from the mock-inoculated (dH_2_O) R0.12 libraries and 0.044% of the reads from the mock-inoculated Dusa® libraries mapped back to the *P. cinnamomi* genome.

**Figure 1:**
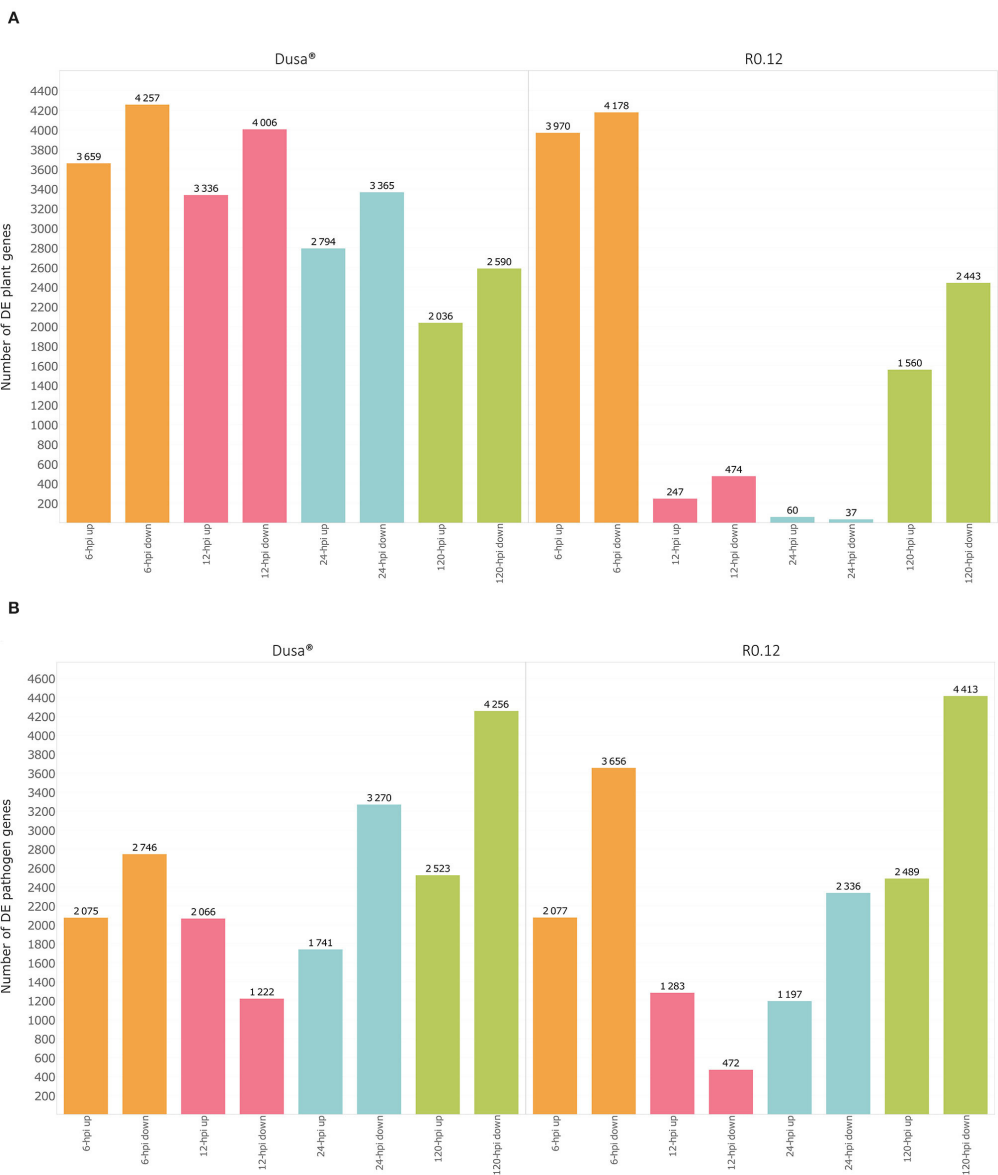
Graphical representation of differentially expressed genes (DEGs) over time in both a susceptible (R0.12) and a partially resistant (Dusa®) rootstock inoculated with *Phytophthora cinnamomi*. **(A)** Total number of significantly up- [log_2_(fold change) > 1, *p* = 0.05] and downregulated avocado genes [log_2_(fold change) < −1, *p* = 0.05] in *P. cinnamomi-*inoculated rootstocks as compared to mock-inoculated controls, shown at 6, 12, 24, and 120 h post inoculation (hpi) in both partially resistant and susceptible avocado rootstocks. **(B)** Total number of significantly up- [log_2_(fold change) > 1, *p* = 0.05] and downregulated *P. cinnamomi* genes [log_2_(fold change) < −1, *p* = 0.05] in inoculated avocado rootstocks as compared to *in vitro* mycelial controls, over time in both Dusa® and R0.12.

Gene expression data were obtained for up to 32,383 Dusa®, 32,588 R0.12, and 16,985 *P. cinnamomi* genes, depending on the sample. When comparing the 6, 12, 24, and 120 hpi Dusa®samples to the mock-inoculated samples, 7,916, 7,342, 6,159 and 4,626 *P. americana* DEGs were observed at each time point, respectively (Figure 1A; Supplementary Table 3). In the R0.12 6, 12, 24, and 120 hpi samples, 8,148, 721, 97, and 4,003 *P. americana* DEGs were observed, respectively, when compared to the mock-inoculated samples (Figure 1A; Supplementary Table 4). When considering *P. cinnamomi*, 4,821, 3,288, 5,011, and 6,779 genes were differentially expressed (DE) in the 6, 12, 24, and 120 hpi Dusa® inoculated samples compared to the mycelial controls (Figure 1B; Supplementary Table 5). Similarly, when compared to the mycelial controls, the 6, 12, 24, and 120 hpi R0.12 samples yielded 5,733, 1,755, 3,533, and 6,902 *P. cinnamomi* DEGs, respectively (Figure 1B; Supplementary Table 6). The differences in log_2_(fold change) between rootstocks, compared at each time point, were also obtained for both *P. americana* (Supplementary Table 7) and *P. cinnamomi* genes (Supplementary Table 8).

**Figure 2:**
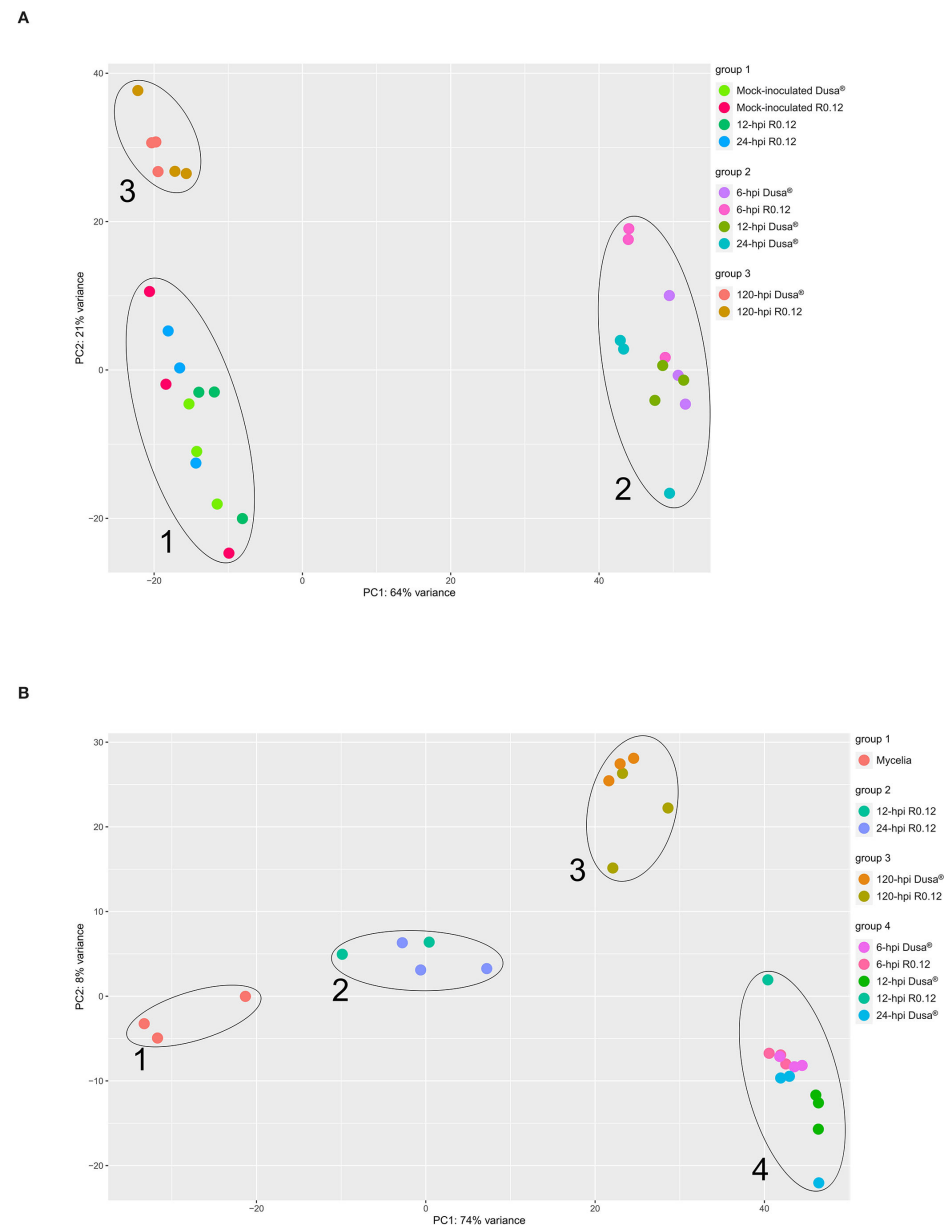
Principal component analysis (PCA) representing variation in gene expression. Expression values were obtained following differential gene expression analyses and variance stabilized transformation. Principal component 1 (PC1) and PC2 were arbitrarily assigned to either axis and represent the total representative variation between samples along that axis. Samples that cluster together were grouped and labeled accordingly. **(A)** *Persea americana* genes over time, following inoculation with *Phytophthora cinnamomi*. The top 500 variably expressed *P. americana* genes across all samples were used for PCA. Samples include both partially resistant (Dusa®) and susceptible (R0.12) *P. americana* rootstocks that were either mock-inoculated (dH_2_O) or inoculated with *P. cinnamomi* and harvested at 6, 12, 24, or 120 h post inoculation (hpi). **(B)** *Phytophthora cinnamomi* genes over time, following inoculation of *Persea americana*. The top 500 variably expressed *P. cinnamomi* genes across all samples were used for PCA. Samples include *in vitro P. cinnamomi* mycelial controls and *in planta* samples, partially resistant (Dusa®) and susceptible (R0.12) *P. americana* rootstocks that were inoculated with *P. cinnamomi* and harvested at 6, 12, 24, or 120 h post inoculation (hpi).

Principal component analyses (PCAs) were performed using the VST-normalized expression data of the top 500 variable genes from either *P. americana* or *P. cinnamomi*. The PCAs were performed to visualize and group the sample libraries in a way conducive to gaining a broad perspective on similarities and differences. For *P. americana* top variable genes, the PCAs identified three distinct sample groups (Figure 2A). The first group contained representative points for mock-inoculated samples from both rootstocks and inoculated R0.12 samples at 12 and 24 hpi. The second group was composed exclusively of early time-point samples from both Dusa® (6, 12, and 24 hpi) and R0.12 (6 hpi). The third group contained only late time-point samples (120 hpi) from both R0.12 and Dusa®. Variance across the first principal component axis, which separates groups 1 and 3 from group 2, represented the largest variation between samples at 64%, while variance across the second principal component axis, separating groups 1 and 2, was only 21%. For *P. cinnamomi* top variable genes, The PCAs resulted in four distinct sample groups (Figure 2B). Group 1 represented *in vitro* mycelia samples only, while group 2 contained all but one sample of R0.12 at 12 and 24 hpi. Group 3 contained representatives from only the late time-point samples (120 hpi) in both rootstocks. Group 4 contained the early time-point samples for Dusa® (6, 12, and 24 hpi) and R0.12 (6 hpi) along with one 12 hpi R0.12 sample. Variance across the first principal component axis accounted for the most variation between groups at 74%, exemplified by groups 1 and 4 occupying opposite ends of the axis. Variance across the second principal component axis accounted for only 8% of the difference between samples, largely distinguishing group 3 from the rest.

**Figure 3:**
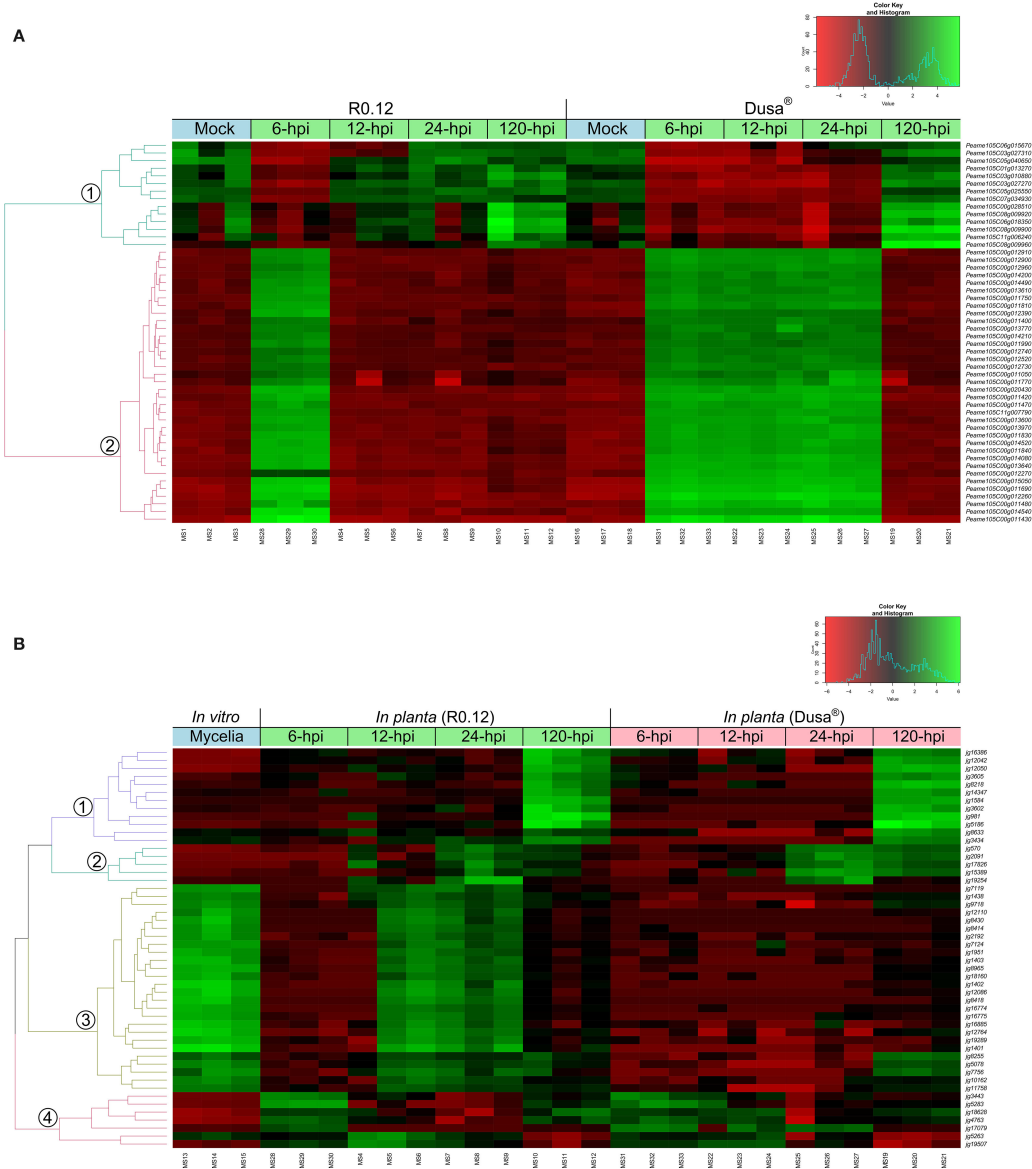
Hierarchical clustering of the top 50 variably expressed *Persea americana* and *Phytophthora cinnamomi* genes. Expression values were obtained following differential gene expression analyses and variance stabilized transformation. The green color represents upregulation, while red indicates downregulation, the scale of which is indicated by the intensity of the color. Gene identifiers are located on the right of the heatmap. Dual RNA-seq library references are indicated along the bottom of the heatmap. Sample clustering is represented by a dendrogram on the left of the heatmap that is color-coded and labeled accordingly. **(A)** Top 50 variably expressed *P. americana* genes across all samples were visualized by hierarchical clustering. Samples include both partially resistant (Dusa®) and susceptible (R0.12) *P. americana* rootstocks that were either mock-inoculated (dH_2_O) or inoculated with *P. cinnamomi* and harvested at 6, 12, 24, or 120 h post inoculation (hpi), indicated along the top of the heatmap. **(B)** Top 50 variably expressed *P. cinnamomi* genes across all samples were visualized by hierarchical clustering. The green color represents upregulation, while red indicates downregulation, the scale of which is indicated by the intensity of the color. Samples include *in vitro P. cinnamomi* mycelial controls and *in planta* samples, partially resistant (Dusa®) and susceptible (R0.12) *P. americana* rootstocks that were inoculated with *P. cinnamomi* and harvested at 6, 12, 24, or 120 h post inoculation (hpi), indicated along the top of the heatmap.

Hierarchical clustering of the top 50 variable genes in both *P. americana* and *P. cinnamomi* was also performed to broadly visualize the differences in gene expression between samples. The dendrogram of the top 50 variable genes in *P. americana* showed two distinct groups of genes with approximately opposite patterns of expression (Figure 3A). The first smaller group contained genes that were expressed to a greater degree in the mock-inoculated samples, 12, 24, and 120 hpi R0.12 samples, and 120 hpi Dusa® samples. Genes in the second larger group displayed greater levels of expression in the 6 hpi R0.12 and 6, 12 and 24 hpi Dusa® samples. In *P. cinnamomi*, four groups of genes with varying expressions were observed (Figure 3B). The first group contained genes that were expressed highest at 120 hpi in both rootstocks, while the second group was expressed highest at 24 and 120 hpi, although to a greater degree in Dusa®. The third group's genes were expressed to the greatest degree in mycelial samples and at 12 and 24 hpi in R0.12 but not Dusa®. Lastly, genes in group 4 were expressed largely at 6 and 120 hpi in R0.12 and 6 and 12 hpi in Dusa®.

### Temporal Cluster Analyses

#### P. americana

**Figure 4:**
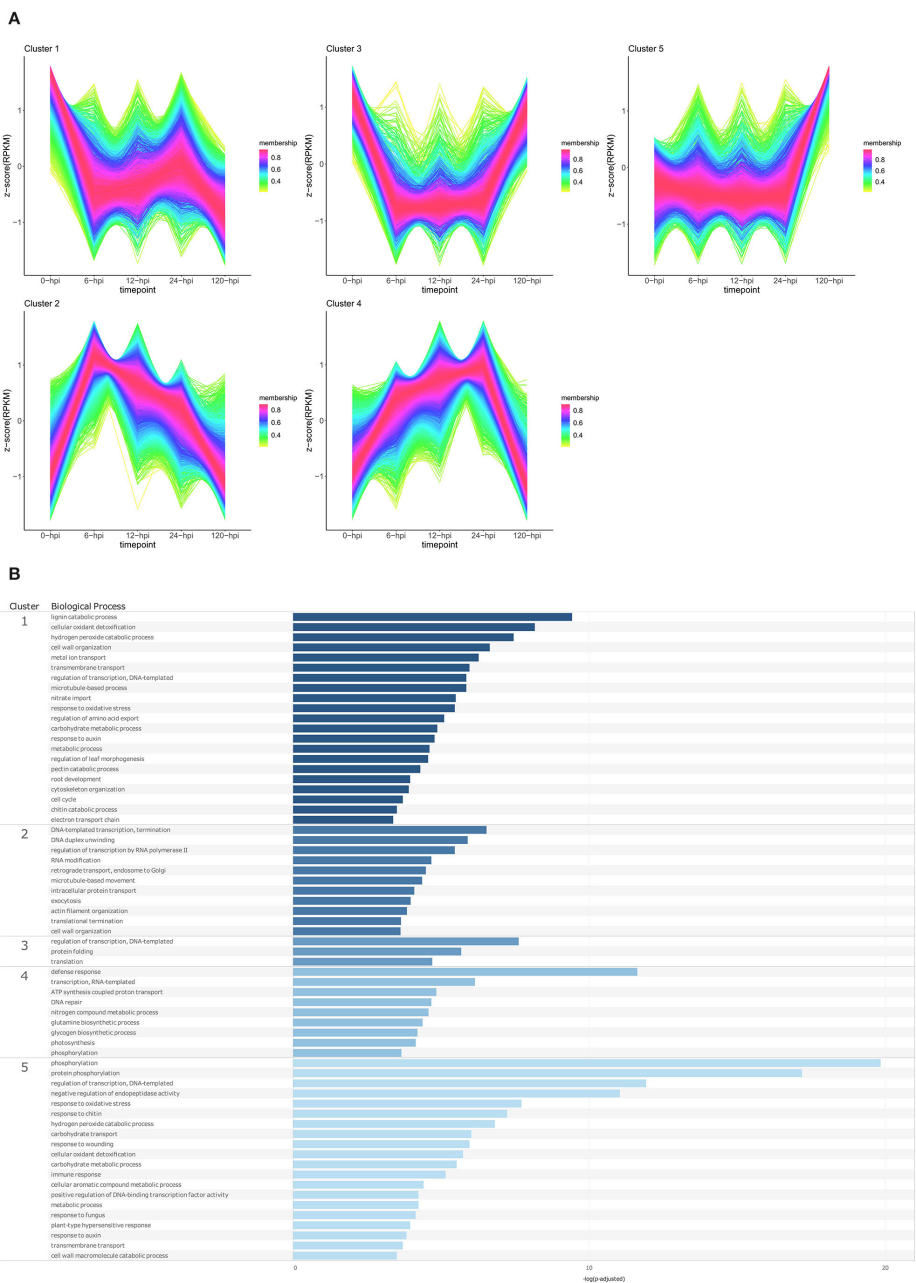
Temporal clustering and Gene Ontology enrichment of *Persea americana* differentially expressed genes (DEGs) in Dusa® following inoculation with *Phytophthora cinnamomi*. **(A)** Sample libraries from the partially resistant *P. americana* rootstock Dusa® following inoculation with *P. cinnamomi* or mock inoculation (dH_2_O) were used in clustering analyses. DEG Z-scores, based on reads per kilobase million (RPKM) normalized read counts, were calculated and used for clustering analyses using the fuzzy c-means (Futschik and Carlisle, 2005) in the R package TCseqv1.16.0 (Wu and Gu, 2021). Inoculated samples included 6, 12, 24, and 120 h post inoculation (hpi) and are indicated on the x-axis, with the mock-inoculated samples indicated as 0 hpi. Membership scores produced by the fuzzy c-means algorithm are color-coded and indicated to the right of each cluster. **(B)** Member genes from each cluster were subjected to Gene Ontology (GO) enrichment, and significantly overrepresented GO terms, with adjusted *p*-value < 0.05 Benjamini-Hochberg false discovery rate, were reduced in REVIGO (Supek et al., 2011). The cluster from which the terms originate is indicated on the leftmost side of the Figure, followed by the biological process GO term. The overrepresented *p*-value of each GO term is indicated along the x-axis as –log_10_ (*p*-adjusted).

**Figure 5:**
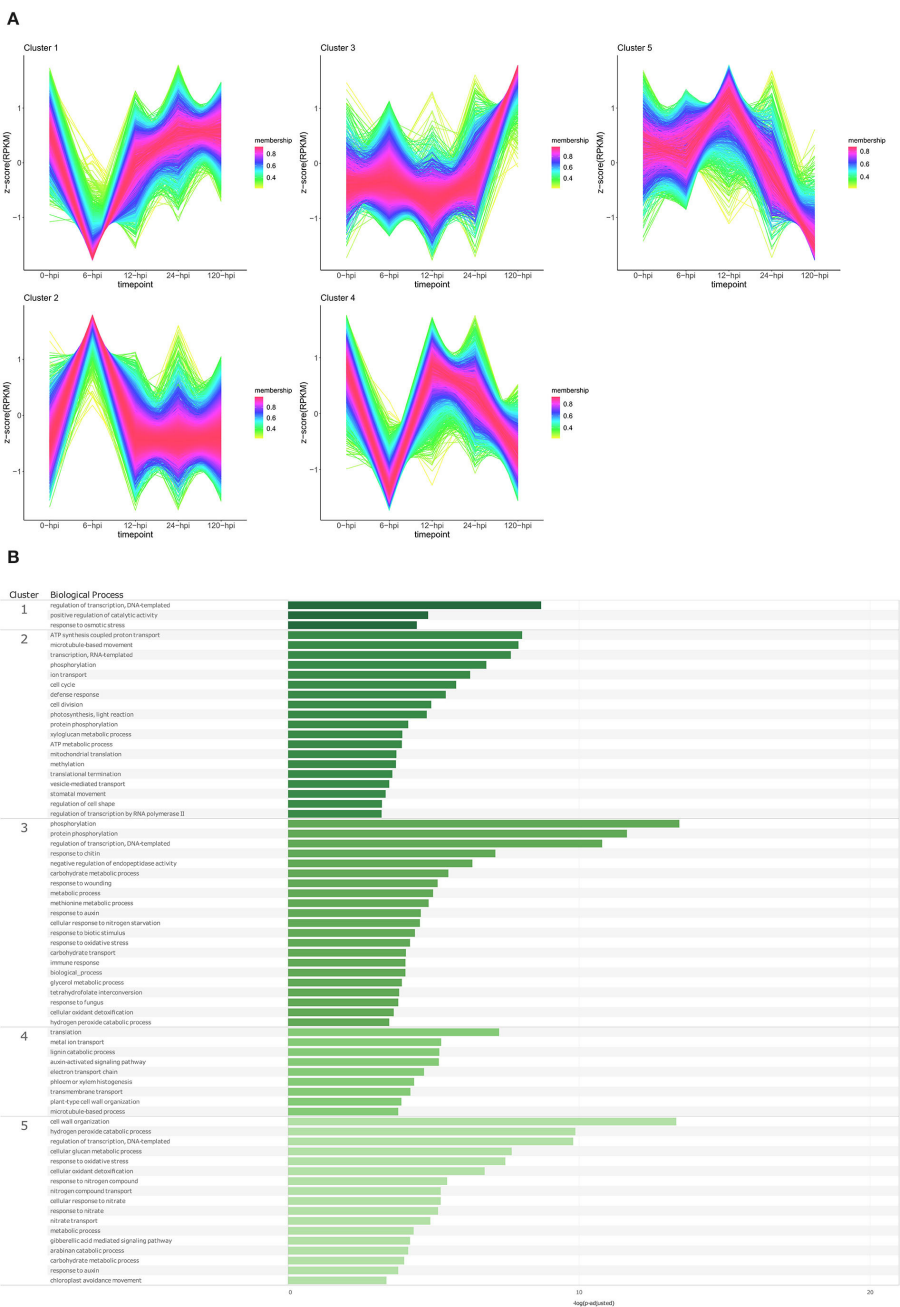
Temporal clustering and Gene Ontology enrichment of *Persea americana* differentially expressed genes (DEGs) in R0.12 following inoculation with *Phytophthora cinnamomi*. **(A)** Sample libraries from the susceptible *P. americana* rootstock R0.12 following inoculation with *P. cinnamomi* or mock inoculation (dH_2_O) were used in clustering analyses. DEG Z-scores, based on reads per kilobase million (RPKM) normalized read counts, were calculated and used for clustering analyses using the fuzzy c-means (Futschik and Carlisle, 2005) in the R package TCseqv1.16.0 (Wu and Gu, 2021). Inoculated samples included 6, 12, 24, and 120 ho post inoculation (hpi) and are indicated on the x-axis, with the mock-inoculated samples indicated as 0 hpi. Membership scores produced by the fuzzy c-means algorithm are color-coded and indicated to the right of each cluster. **(B)** Member genes from each cluster were subjected to Gene Ontology (GO) enrichment and significantly overrepresented GO terms, with adjusted *p*-value < 0.05 Benjamini-Hochberg false discovery rate, were reduced in REVIGO (Supek et al., 2011). The cluster from which the terms originate is indicated on the leftmost side of the Figure, followed by the biological process GO term. The overrepresented *p*-value of each GO term is indicated along the x-axis as –log_10_ (*p*-adjusted).

Temporal cluster analyses were performed to separate the *P. americana* genes based on their expression in mock-inoculated samples (0 hpi), and over time following *P. cinnamomi* inoculation. Five clusters with distinct temporal patterns were obtained for both Dusa® and R0.12 (Figures 4A, 5A; Supplementary Table 9). In Dusa®, cluster 1 represented genes with reduced expression at 6, 12, and 24 hpi followed by further reduction at 120 hpi. Overrepresented biological process (BP) GO terms resulting from this cluster included lignin, pectin, and chitin catabolic processes, several terms related to growth and development such as response to auxin, root development, nitrate import, and several terms related to oxidative stress (Figure 4B; Supplementary Table 10). The expression of genes in cluster 2 increased significantly at 6 hpi, reducing gradually at each subsequent time point until the expression at 120 hpi closely resembled that of the mock-inoculated samples (0 hpi). Overrepresented BP terms from genes in this cluster included transcription, translation, RNA modification, intracellular protein transport, and exocytosis. Cluster 3 contained genes that were expressed at their lowest levels at 6, 12, and 24 hpi, while the expression at 0 and 120 hpi seemed similar. Gene ontology enrichment of genes in this cluster only yielded three BP terms, DNA-templated regulation of transcription, protein folding, and translation. Cluster 4 contained genes that increased in expression during the early time points, peaking at 24 hpi, followed by a drop slightly below baseline expression at 120 hpi. Enriched BP terms from this cluster included phosphorylation, defense response, DNA repair, RNA-templated transcription, and several terms related to metabolism and photosynthesis. Expression of genes in cluster 5 remained largely stable across the mock-inoculated and early time-point samples, followed by a definitive increase at 120 hpi. Overrepresented BP terms from genes in this cluster included phosphorylation, negative regulation of endopeptidase activity, immune response, plant type hypersensitive response, cellular aromatic compound metabolic process, response to auxin, response to chitin, wounding and fungi, and several terms related to oxidative stress.

In the susceptible rootstock R0.12, temporal cluster analyses yielded five clusters that were objectively different to those obtained for Dusa® (Figure 5A; Supplementary Table 9). Genes in cluster 1 showed substantially decreased expression at 6 hpi, however, at 12, 24, and 120 hpi, their expression could be matched to the expression in the mock-inoculated samples. GO enrichment of genes in this cluster resulted in a limited list of BP terms, DNA-templated regulation of transcription, positive regulation of catalytic activity, and response to osmotic stress (Figure 5B; Supplementary Table 11). Cluster 2 showed a trend opposite to that of cluster 1, with genes from this cluster displaying increased expression at 6 hpi, followed by a return to baseline at 12 hpi. Overrepresented BP terms obtained from genes in this cluster included phosphorylation, defense response, several terms related to transcription, translation, growth and development, metabolism, and photosynthesis. The expression of genes in cluster 3 remained relatively unchanged in the mock-inoculated and early time-point samples, with a distinctive increase at 120 hpi. Several enriched BP terms were obtained from genes in this cluster, including phosphorylation, response to chitin, wounding and fungi, response to auxin, cellular response to nitrogen starvation, negative regulation of endopeptidase activity, and several terms related to metabolism and oxidative stress. Cluster 4 contained genes that experienced a decrease in expression at 6 hpi, an increase back to baseline levels at 12 hpi, and a subsequent progressive decrease at 24 and 120 hpi. Biological process terms significantly enriched in this group included translation, auxin activated signaling pathway, lignin catabolic process, plant cell wall organization, and phloem/xylem histogenesis. Lastly, the expression of genes in cluster 5 was equivalent at 0 and 6 hpi, noticeably increased at 12 hpi, and decreased to well below baseline levels at 120 hpi. Enriched BP terms in this cluster included gibberellic acid-mediated signaling pathway, response to auxin, several terms related to oxidative stress and, interestingly, many terms related to growth and metabolism. The results of GO enrichment analyses for *P. americana* genes at individual time points are also available for both Dusa® and R0.12 (Supplementary Figures 1, 2; Supplementary Tables 12–15).

The member genes, expression profiles, and GO profile of each cluster in both rootstocks were also compared to one another. Two clusters from each rootstock were highly similar, cluster 2 from both Dusa® and R0.12 with 2,015 shared genes and clusters 5 from Dusa® and 3 from R0.12 that shared 1,652 genes (Supplementary Table 16). The expression of genes in cluster 2 from both rootstocks peaked at 6 hpi, although in R0.12, the expression returned to mock-inoculated levels at 12 hpi, but in Dusa® the expression only returned to mock-inoculated levels at 120 hpi. Interestingly, the number of shared genes between clusters 2 from R0.12 and 4 from Dusa® was also substantial, but in Dusa®, the average gene expression rose steadily from 6- to 24 hpi. Likely the most similar were clusters 5 from Dusa® and 3 from R0.12, in which the gene expression remained mostly unchanged until 120 hpi. Additionally, in both clusters, enriched BP terms were near identical. Cluster 1 from Dusa®seemed most like cluster 4 from R0.12 because of the similarity of some BP terms, expression at 0, 6, and 120 hpi, and shared member gene identity. Still, some similarities were seen when comparing cluster 1 from Dusa® to cluster 5 from R0.12. However, the expression of genes from cluster 1 in Dusa® only decreased over time, whereas in R0.12, the expression peaked at 12 hpi and decreased thereafter. Lastly, cluster 3 in Dusa® seemed to resemble both clusters 1 and 4 in R0.12 to some degree. In all three of these clusters, the gene expression decreased at 6 hpi while remaining low at 12 and 24 hpi in Dusa® but not in R0.12.

#### P. cinnamomi

**Figure 6:**
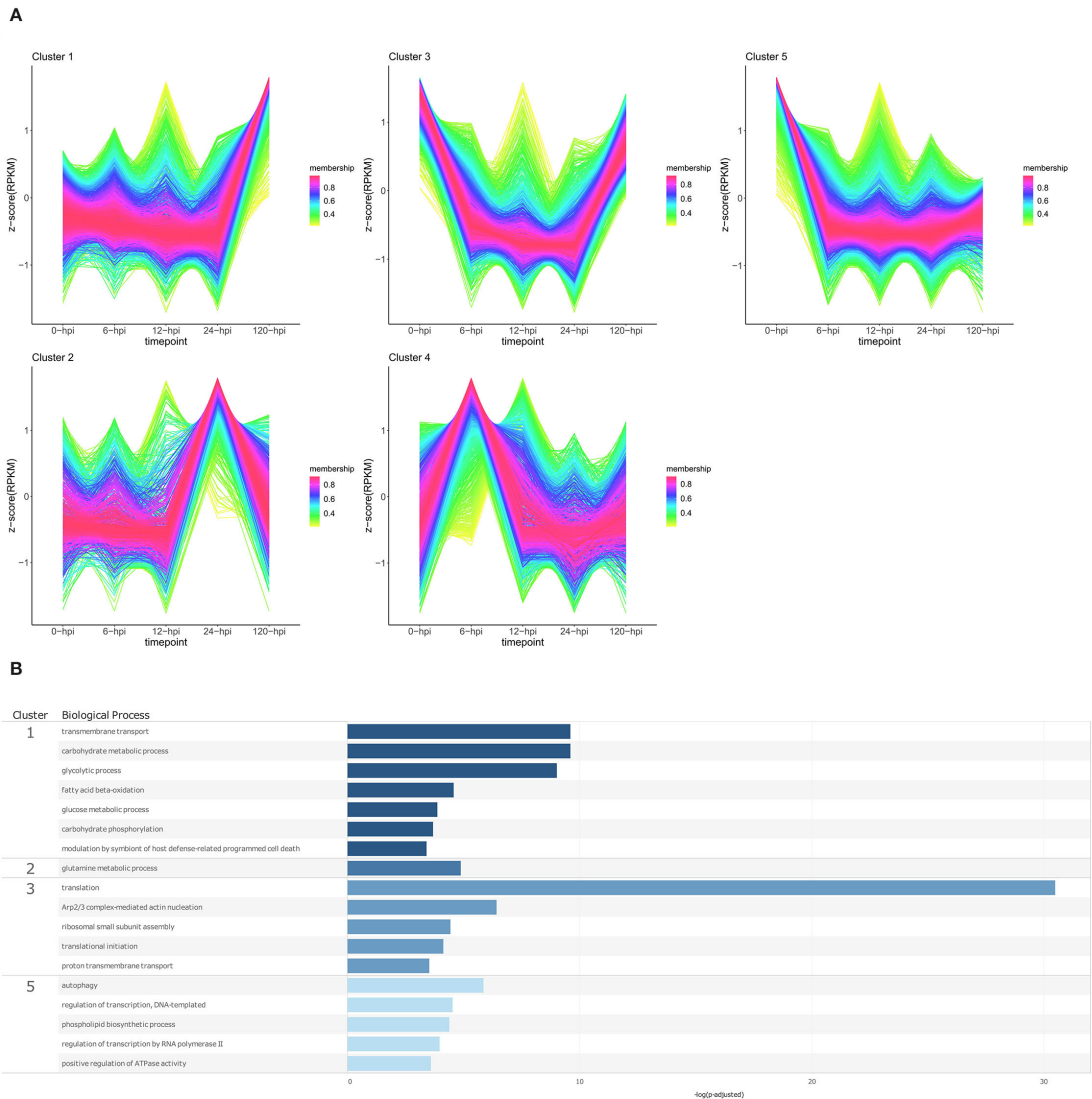
Temporal clustering and Gene Ontology enrichment of *Phytophthora cinnamomi* differentially expressed genes (DEGs) following inoculation of the *Persea americana* rootstock Dusa®. **(A)** Sample libraries from *in vitro* mycelial controls and the partially resistant *P. americana* rootstock Dusa®inoculated with *P. cinnamomi* were used in clustering analyses. DEG Z-scores, based on reads per kilobase million (RPKM) normalized read counts, were calculated and used for clustering analyses using the fuzzy c-means (Futschik and Carlisle, 2005) in the R package TCseqv1.16.0 (Wu and Gu, 2021). Inoculated samples included 6, 12, 24, and 120 h post inoculation (hpi) and are indicated on the x-axis, with the mock-inoculated samples indicated as 0 hpi. Membership scores produced by the fuzzy c-means algorithm are color-coded and indicated to the right of each cluster. **(B)** Member genes from each cluster were subjected to Gene Ontology (GO) enrichment and significantly overrepresented GO terms, with adjusted *p*-value < 0.05 Benjamini-Hochberg false discovery rate, were reduced in REVIGO (Supek et al., 2011). The cluster from which the terms originate is indicated on the left most side of the Figure, followed by the biological process GO term. The overrepresented *p*-value of each GO term is indicated along the x-axis as –log_10_ (*p*-adjusted).

**Figure 7:**
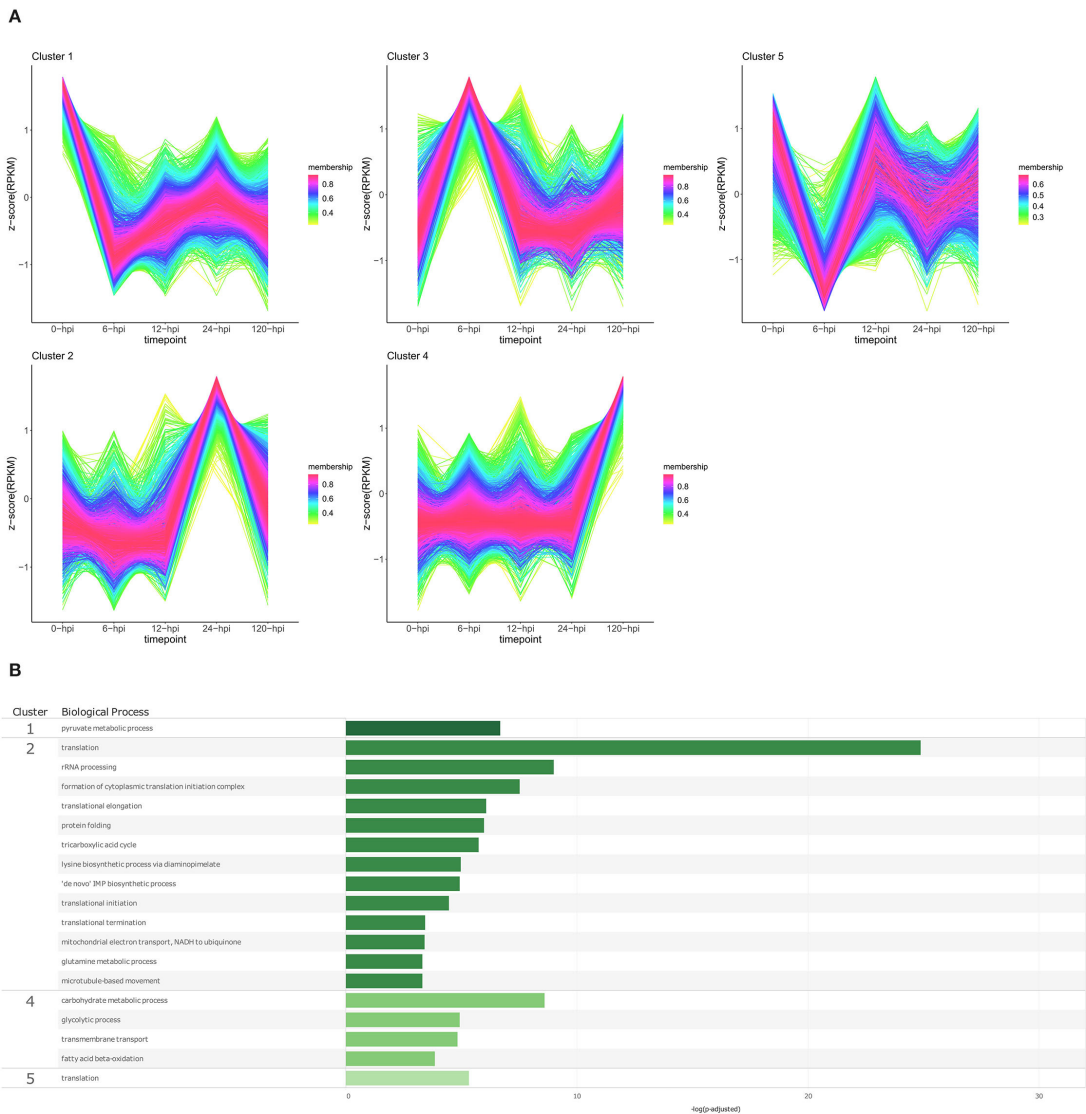
Temporal clustering and Gene Ontology enrichment of *Phytophthora cinnamomi* differentially expressed genes (DEGs) following inoculation of the *Persea americana* rootstock R0.12. **(A)** Sample libraries from *in vitro* mycelial controls and the susceptible *P. americana* rootstock R0.12 inoculated with *P. cinnamomi* were used in clustering analyses. DEG Z-scores, based on reads per kilobase million (RPKM) normalized read counts, were calculated and used for clustering analyses using the fuzzy c-means (Futschik and Carlisle, 2005) in the R package TCseqv1.16.0 (Wu and Gu, 2021). Inoculated samples included 6, 12, 24, and 120 h post inoculation (hpi) and are indicated on the x-axis, with the mock-inoculated samples indicated as 0 hpi. Membership scores produced by the fuzzy c-means algorithm are color-coded and indicated to the right of each cluster. **(B)** Member genes from each cluster were subjected to gene ontology (GO) enrichment, and significantly overrepresented GO terms, with adjusted *p*-value < 0.05 Benjamini-Hochberg false discovery rate, were reduced in REVIGO (Supek et al., 2011). The cluster from which the terms originate is indicated on the left most side of the Figure, followed by the biological process GO term. The overrepresented *p*-value of each GO term is indicated along the x-axis as –log_10_ (*p*-adjusted).

Temporal cluster analyses were performed to separate *P. cinnamomi* genes based on their expression in *in vitro* cultured mycelia, and over time following inoculation of a partially resistant (Dusa®) and a susceptible (R0.12) *P. americana* rootstock (Figures 6A, 7A; Supplementary Table 17). Following inoculation of Dusa®, the expression of *P. cinnamomi* genes belonging to cluster 1 remained similar to that of mycelia (0 hpi) at early time-points, while at 120 hpi, the expression increased considerably. Overrepresented BP terms resulting from genes in this cluster included transmembrane transport, modulation by symbiont of host defense-related PCD, and several terms related to metabolism (Figure 6B; Supplementary Table 18). The expression of genes in cluster 2 showed a single peak at 24 hpi, and only one enriched BP term was obtained for this cluster, glutamine metabolic process. Genes in cluster 3 showed lower expression at 6, 12, and 24 hpi when compared to mycelia, and at 120 hpi, and biological process terms associated with genes from this cluster were mostly related to translation. The expression of genes in cluster 4 was higher at 6 hpi than at other time points or that of mycelia; however, no enriched BP terms were recovered for this cluster. Cluster 5 contained genes that were expressed at much lower levels *in planta* than in mycelia, and BP terms associated with this cluster included autophagy, transcription, phospholipid biosynthesis, and positive regulation of ATPase activity.

Temporal cluster analyses of R0.12 yielded five clusters with profiles similar to those seen in Dusa®. In R0.12, cluster 1 included genes that are expressed highest in the *in vitro* mycelia samples rather than *in planta* (Figure 7A; Supplementary Table 17). The only enriched BP term obtained from genes in this cluster was pyruvate metabolic process (Figure 7B; Supplementary Table 19). Cluster 2 included genes that displayed high expression at 24 hpi, while at all the other time points, levels were equivalent. Overrepresented BP terms associated with genes from this cluster included rRNA processing, protein folding, and several terms related to translation and metabolism. Genes that formed part of cluster 4 were expressed at much higher levels at 120 hpi than at any other time-point or in mycelia. Enriched BP terms resulting from genes in cluster 4 included transmembrane transport and several terms related to metabolism. The expression of genes in cluster 5 was substantially lower at 6 hpi when compared to mycelia; at 12 hpi, levels were equivalent to mycelia followed by slightly lower levels at 24 and 120 hpi. The only BP term enriched using genes in this cluster was translation. The results of GO enrichment analyses for *P. cinnamomi* genes at individual time points following inoculation of both Dusa® and R0.12 are also available (Supplementary Figures 3, 4; Supplementary Tables 20–23)

The member genes, expression profiles, and GO terms for each *P. cinnamomi* temporal cluster was compared between the rootstocks (Supplementary Table 24). Although near-identical expression profiles could be identified for each cluster from either rootstock, member gene lists were only highly similar for two clusters from each rootstock, cluster 1 from Dusa® and cluster 4 from R0.12, and cluster 5 from Dusa® and cluster 1 from R0.12, which shared 1,120 and 1595 genes, respectively. Cluster 3 in Dusa® shared 471 genes with cluster 5 in R0.12; they also shared enriched BP terms related to translation. Meanwhile, cluster 4 in Dusa® and cluster 3 in R0.12 shared 552 genes, neither of which contained any enriched BP terms. Cluster 2 from both rootstocks contained genes that were expressed highest at 24 hpi, although they only shared 231 genes. For perspective, cluster 2 from R0.12 shared a similar number of genes with clusters 1, 3, and 5 from Dusa®.

#### *P. cinnamomi* Virulence Factors

Following cluster analyses, the corresponding predicted proteins from *P. cinnamomi* genes in each cluster were compared to the PHI database to identify important virulence determinants at defined time points (Supplementary Tables 25, 26). On average, 33% of *P. cinnamomi* proteins from described clusters, following the inoculation of Dusa® and R0.12, matched to the PHI database sequences with an E-value < 1.0e^−5^ (Table 1). Overall, 19 different types of effectors were identified, 10 of which were well-documented in *Phytophthora* species; RxLR effector proteins were predominant, followed by elicitins, necrosis, and ethylene-inducing peptide 1 (Nep1)-like proteins (NLPs),) glucanase inhibitor proteins (GIPs), and EPIs, among others (Table 2). Of the 40 unique RxLRs identified, only 16 were identified in both rootstocks. A similar observation was made for all identified *Phytophthora* effectors except for XEG1 and *Phytophthora cactorum*-Fragaria (PcF), indicating a distinctive compliment of effectors was used during the infection of each rootstock.

**Table 1:** Summary statistics following comparison of temporal cluster member protein sequences to the Pathogen Host Interactions (PHI) database.

**Rootstock**	**Cluster**	**Cluster peak**	**Total query sequences**	**Unique PHI-base matches**	**Percentage query matches**
Dusa®	1	120 hpi	2,191	794	36,24%
	2	24 hpi	848	296	34,91%
	3	n/a	2,260	694	30,71%
	4	6 hpi	1,434	476	33,19%
	5	n/a	3,685	1,139	30,91%
R0.12	1	n/a	2,070	798	31,01%
	2	24 hpi	1,383	590	34,20%
	3	6 hpi	1,310	665	35,95%
	4	120 hpi	1,815	785	34,93%
	5	n/a	1,262	432	28,05%

*Blastp comparison of query sequences to PHI-base was performed with an Expect value (E) cutoff of 1.0e^−5^. A cluster peak was considered when the expression of member genes exceeded that of baseline (0 hpi) at any other time point. hpi, Hours post inoculation*.

**Table 2:** Number and types of known *Phytophthora* effectors identified following the comparison of temporal cluster member protein sequences to the Pathogen-Host Interactions (PHI) database.

	**Dusa^®^**				**R0.12**					
**Effector**	**6 hpi**	**24 hpi**	**120 hpi**	**Total**	**6 hpi**	**24 hpi**	**120 hpi**	**Total**	**Unique**	**Shared**
RxLR	7	9	14	30	1	19	6	26	40	16
Elicitin	0	0	12	12	1	3	7	11	14	9
NLP	0	1	9	10	2	3	6	11	14	7
GIP	0	4	7	11	0	4	1	8	11	8
EPI	0	4	6	10	0	3	3	6	11	5
XEG1	0	0	6	6	0	2	4	6	6	6
CBEL	4	1	1	6	3	1	1	5	6	5
CRN	1	1	0	2	0	1	2	3	5	0
PcF	0	0	1	1	0	1	0	1	1	1

*Necrosis and an ethylene-inducing peptide 1 (Nep1)-like protein (NLP), glucanase inhibitor protein (GIP), extracellular protease inhibitor (EPI), xyloglucan-specific endo-beta-1,4-glucanase 1 (XEG1), cellulose-binding elicitor lectin (CBEL), crinkling and necrosis (CRN), and Phytophthora cactorum-Fragaria (PcF). A cluster peak was considered when the expression of member genes exceeded that of baseline (0 hpi) at any other time point. hpi, Hours post inoculation*.

When only considering matches to the effector proteins from the PHI database, cluster 4 from Dusa® and cluster 3 from R0.12, which both peak at 6 hpi, contain several notable matches. This comparison identified several *P. cinnamomi* genes, following the inoculation of either rootstock, which encoded for proteins with similarity to CBEL and RxLR effector proteins from other *Phytophthora* species (Supplementary Tables 25, 26). In both rootstocks, more unique predicted CBELs, four in Dusa® and three in R0.12, were identified in the 6 hpi peak clusters than in any of the other clusters. Contrastingly, the lowest number of predicted proteins with similarity to RxLRs was identified in these clusters as compared to the 24 and 120 hpi peak clusters. Here, only one predicted RxLR was identified in R0.12 (jg4048), whereas seven were identified in Dusa®. Interestingly, in inoculated Dusa®, there were also matches to a crinkling and necrosis (CRN) protein from *Phytophthora sojae*. In R0.12, similarities to an elicitin from *Phytophthora infestans* and an NLP from *Phytophthora capsici* were found.

The expression of *P. cinnamomi* genes in cluster 2 from both Dusa® and R0.12 peaked at 24 hpi. In these clusters, as might be expected, far fewer predicted proteins with similarity to CBEL were identified. However, following the inoculation of both rootstocks, several genes that code for proteins with similarity to GIP2 in *P. sojae* and EPIs from *P. infestans* were identified. It was noted, however, that the compliment of EPIs and GIPs present in either rootstock was distinctly different, with less than half of the proteins being shared between clusters. Further differences between the rootstocks included the presence of three proteins with similarity to elicitins that were present in R0.12 but not in Dusa®. Likewise, 19 proteins with similarity to RxLRs from *P. sojae* and *P. infestans* were identified in R0.12, while only nine were identified in Dusa®, contrasting with the observation made at 6 hpi. Three predicted NLP-like proteins were also identified following the inoculation of R0.12, while only one was identified in Dusa®. Surprisingly, the predicted protein sequence jg108, which showed similarity to the phytotoxic protein PcF, was found in cluster 2 of R0.12 but not in Dusa®.

Otherwise, the repertoire of effector proteins identified in cluster 4 from R0.12, in which the gene expression peaked at 120 hpi, was significantly less diverse when compared to R0.12 cluster 2; cluster 4 contained only six predicted RxLRs, three EPIs, one GIP, and four XEGs. In the analogous cluster from Dusa®, cluster 1, there were 14 predicted RxLRs, six EPIs, seven GIPs, and six XEGs. Elicitins were also identified in Dusa® for the first time, totaling 12, whereas seven were identified in R0.12. In Dusa® and R0.12, the total number of predicted NLP effector proteins also increased to nine and six, respectively. Notably, jg1926, a predicted NLP protein with high similarity to necrosis-inducing *Phytophthora* protein 1 (NPP1) from *Phytophthora parasitica*, was identified in the cluster from R0.12 but not in Dusa®. Intriguingly, the same predicted CBEL, jg11698, was identified in both Dusa® and R0.12 at this time point. In contrast to the observations made on clusters 2 from both rootstocks, PcF was identified in Dusa® cluster 1 and not in R0.12 cluster 4.

#### *P. americana* Defense Responses

Mercator was used to assign the predicted *P. americana* proteins to MapMan bins. Mercator3 successfully annotated 54.2% of the 47,687 predicted *P. americana* protein sequences, assigning 32.39% to the bins and occupying 94.25% of the available bins (Supplementary Table 27). Mercator4 successfully annotated 47.52% of the predicted *P. americana* predicted sequences, assigning 33.59% to the bins and occupying 94.34% of the available bins (Supplementary Table 28). Mercator 3 annotation conducted for further investigations because of the higher number of annotated *P. americana* proteins, 25,845 vs. 22,661. DEGs were then visualized in MapMan to assist in pathway identification and comparison.

However, because of the significant contrast in the number of DEGs between Dusa® and R0.12 at 12 and 24 hpi (13,501 and 818, respectively), the list of top 100 variably expressed genes was first used to filter potential candidate genes and pathways for further investigation (Supplementary Table 29). To further reduce this list, only genes known to play a direct role in plant defense or stress responses were kept, while genes involved in biological processes, e.g., photosynthesis and electron transport, were omitted. The filtered list included 35 genes involved in the biosynthesis and regulation of some phytohormones, PR protein coding genes, protease inhibitors, proteases, several extensins, and a wall-associated kinase (WAK), among others (Table 3). Given the limited knowledge of receptor-like kinases and apoplastic proteases in *P. americana*, we decided to focus the further analysis on these two groups of proteins.

**Table 3:** Filtered list of the most variably expressed defense- and stress-related genes between R0.12 and Dusa® following *P. cinnamomi* inoculation.

**Gene ID**	**Brief description**	**Predicted protein description (NCBI)**
*Peame105C01g004420*	JA biosynthesis	12-oxophytodienoate reductase 2-like protein [*Cinnamomum micranthum* f. *kanehirae*]
*Peame105C06g015670*	ABA biosynthesis	9-cis-epoxycarotenoid dioxygenase [*Persea americana*]
*Peame105C02g039760*	JA biosynthesis	Allene oxide synthase 1, chloroplastic-like protein [*Cinnamomum micranthum* f. *kanehirae*]
*Peame105C05g040650*	Aquaporin	Putative aquaporin TIP1-2 [*Cinnamomum micranthum* f. *kanehirae*]
*Peame105C02g052930*	Glucosidase	Lysosomal beta glucosidase-like protein [*Cinnamomum micranthum* f. *kanehirae*]
*Peame105C03g027310*	ABA positive regulator	EID1-like F-box protein 3 [*Vitis riparia*]
*Peame105C07g031780*	Germin-like	Putative germin-like protein 2-1 [*Cinnamomum micranthum* f. *kanehirae*]
*Peame105C05g038480*	Germin-like	Putative germin-like protein 2-1 [*Cinnamomum micranthum* f. *kanehirae*]
*Peame105C04g007260*	Chitinase	Chitotriosidase-1-like protein [*Cinnamomum micranthum* f. *kanehirae*]
*Peame105C03g003970*	Chitinase	Acidic mammalian chitinase-like protein [*Cinnamomum micranthum* f. *kanehirae*]
*Peame105C00g011050*	Glycosylation	Glycosyl transferase (glycosyl transferase family 2) [*Trifolium pratense*]
*Peame105C11g000290*	Abiotic stress response	Heavy metal-associated isoprenylated plant protein 3 isoform X1 [*Cinnamomum micranthum* f. *kanehirae*]
*Peame105C06g014320*	Drought response	Homeobox-leucine zipper protein ATHB-12-like protein isoform X1 [*Cinnamomum micranthum* f. *kanehirae*]
*Peame105C03g043980*	JA biosynthesis	Linoleate 13S-lipoxygenase 2-1, chloroplastic-like protein [*Cinnamomum micranthum* f. *kanehirae*]
*Peame105C02g019990*	Protease	Metalloendoprotease 2-MMP-like protein [*Cinnamomum micranthum* f. *kanehirae*]
*Peame105C10g013040*	Protease	Basic 7S globulin [*Cinnamomum micranthum* f. *kanehirae*]
*Peame105C11g018410*	Protease	Aspartyl protease AED3 [*Cinnamomum micranthum* f. *kanehirae*]
*Peame105C08g009900*	Protease inhibitor	gluS.griseus protease inhibitor [*Cinnamomum micranthum* f. *kanehirae*]
*Peame105C08g009920*	Protease inhibitor	Protease inhibitor [*Cinnamomum micranthum* f. *kanehirae*]
*Peame105C00g028510*	Protease inhibitor	Protease inhibitor [*Cinnamomum micranthum* f. *kanehirae*]
*Peame105C08g009960*	Protease inhibitor	gluS.griseus protease inhibitor [*Cinnamomum micranthum* f. *kanehirae*]
*Peame105C08g009990*	Protease Inhibitor	gluS.griseus protease inhibitor [*Cinnamomum micranthum* f. *kanehirae*]
*Peame105C02g036640*	PR-1	Pathogenesis-related protein PRB1-3-like protein [*Cinnamomum micranthum* f. *kanehirae*]
*Peame105C06g018350*	PR-5	Protein P21-like protein [*Cinnamomum micranthum* f. *kanehirae*]
*Peame105C11g006240*	PR-5	Thaumatin-like protein 1 [*Cinnamomum micranthum* f. *kanehirae*]
*Peame105C11g006180*	PR-5	Thaumatin-like protein 1 [*Cinnamomum micranthum* f. *kanehirae*]
*Peame105C06g018290*	PR-5	Pathogenesis related protein-5 [*Cinnamomum micranthum* f. *kanehirae*]
*Peame105C03g027270*	Extensin (kinase)	Extensin-2-like [*Papaver somniferum*]
*Peame105C03g010880*	Extensin (kinase)	Extensin [*Trema orientale*]
*Peame105C05g025550*	Extensin (kinase)	Extensin-2-like [*Cucurbita moschata*]
*Peame105C03g027260*	Extensin (kinase)	Extensin-2-like [*Cucurbita moschata*]
*Peame105C01g013210*	Extensin (kinase)	PREDICTED: repetitive proline-rich cell wall protein-like [*Daucus carota* subsp. *sativus*]
*Peame105C07g012900*	Wall-associated kinase	Wall-associated receptor kinase 2-like protein [*Cinnamomum micranthum* f. *kanehirae*]
*Peame105C03g046420*	Abiotic stress response	Zinc finger protein ZAT10 [*Cinnamomum micranthum* f. *kanehirae*]
*Peame105C05g023890*,	Abiotic stress response	Zinc finger protein ZAT10 [*Cinnamomum micranthum* f. *kanehirae*]

*JA, jasmonic acid; ABA, abscisic acid; PR, pathogenesis-related*.

#### Receptor-Like Kinases

When visualized using MapMan, clear differences in the number of DE receptor-like kinases (RLKs) were apparent, even at 6 and 120 hpi, where R0.12 and Dusa® displayed less differences overall (Table 4). In Dusa®, far more L-type lectin (L-lectin), thaumatin-like protein kinase (TLPK), receptor-like kinase in flowers 3 (RFK3-like), crinkly4-like (CR4-like), proline-rich extensin-like receptor protein kinase (PERK-like), and leaf rust 10 disease-resistance locus receptor-like protein kinase (LRK10-like) coding genes were significantly downregulated at 6, 12, and 24 hpi than those that were upregulated. While at 120 hpi the opposite was observed - far more were significantly upregulated for each than were downregulated. A related distinction could be made between 6 and 120 hpi for L-lectin, TLPK, RFK3, CR4-like, PERK-like, and LRK10-like coding genes in R0.12. However, fewer of these genes, except for CR4-like, were significantly upregulated in R0.12 at 120 hpi when compared to Dusa®.

**Table 4:** Number of predicted receptor-like kinase (RLK) family proteins encoded for by genes that are significantly up- and downregulated in Dusa® or R0.12 following inoculation with *Phytophthora cinnamomi*.

			**Dusa^®^**								**R0.12**							
			**No. upregulated**				**No. downregulated**				**No. upregulated**				**No. downregulated**			
		**Total identified**	**6 hpi**	**12 hpi**	**24 hpi**	**120 hpi**	**6 hpi**	**12 hpi**	**24 hpi**	**120 hpi**	**6 hpi**	**12 hpi**	**24 hpi**	**120 hpi**	**6 hpi**	**12 hpi**	**24 hpi**	**120 hpi**
L-Lectin		81	4%	2%	2%	21%	12%	11%	14%	2%	1%	0%	1%	11%	9%	5%	0%	5%
TLPK		88	6%	2%	2%	19%	9%	6%	7%	1%	7%	0%	0%	9%	5%	6%	0%	1%
WAK-like		98	12%	6%	4%	9%	8%	7%	5%	3%	10%	1%	1%	8%	7%	2%	0%	6%
S-domain		162	17%	12%	13%	15%	5%	3%	2%	4%	22%	0%	0%	12%	4%	3%	0%	1%
RFK3-like		6	0%	0%	0%	33%	33%	33%	17%	0%	0%	0%	0%	17%	17%	17%	0%	0%
PERK-like		25	8%	8%	4%	12%	12%	20%	16%	4%	12%	0%	0%	8%	16%	4%	0%	4%
LRK10-like		105	6%	3%	8%	16%	10%	6%	1%	2%	7%	0%	0%	9%	6%	7%	0%	2%
DUF26		130	13%	9%	13%	18%	5%	5%	4%	2%	21%	1%	1%	15%	6%	1%	0%	2%
CR4-like		10	10%	10%	0%	30%	30%	40%	20%	20%	10%	0%	0%	30%	20%	10%	0%	0%
LysM		16	6%	0%	6%	6%	6%	0%	0%	6%	0%	0%	0%	0%	6%	0%	0%	6%
C-Lectin		1	0%	0%	0%	0%	0%	0%	0%	0%	0%	0%	0%	0%	0%	0%	0%	0%
Extensin		16	0%	0%	0%	6%	13%	13%	13%	13%	0%	0%	0%	6%	13%	6%	0%	13%
Leucine-rich repeat L	Type I	94	13%	13%	11%	2%	2%	2%	1%	7%	18%	1%	0%	3%	3%	2%	0%	0%
Leucine-rich Repeat	Type II	16	6%	19%	13%	13%	6%	6%	6%	6%	6%	6%	0%	6%	13%	0%	0%	13%
	Type III	45	0%	7%	4%	2%	36%	33%	31%	33%	2%	0%	0%	0%	36%	0%	0%	38%
	Type IV	5	0%	0%	0%	0%	0%	0%	0%	40%	0%	0%	0%	0%	0%	0%	0%	40%
	Type V	10	0%	10%	0%	0%	10%	10%	10%	10%	0%	0%	0%	0%	0%	0%	0%	10%
	Type VI	14	0%	0%	7%	7%	7%	0%	0%	29%	0%	0%	0%	7%	0%	0%	0%	14%
	Type VII	9	22%	0%	11%	0%	33%	33%	33%	22%	22%	0%	0%	0%	33%	0%	0%	33%
	Type VIII	71	48%	41%	34%	20%	6%	6%	3%	6%	46%	3%	0%	10%	8%	4%	0%	3%
	Type IX	6	17%	0%	0%	0%	0%	17%	0%	17%	17%	17%	0%	0%	17%	0%	0%	33%
	Type X	34	15%	18%	24%	9%	3%	3%	3%	9%	24%	0%	0%	12%	3%	0%	0%	3%
	Type XI	65	6%	3%	5%	8%	25%	26%	18%	31%	9%	0%	0%	2%	22%	0%	0%	32%
	Type XII	219	12%	10%	13%	5%	3%	3%	2%	3%	18%	1%	0%	6%	4%	1%	0%	4%
	Type XIII	14	14%	7%	21%	0%	0%	0%	0%	7%	21%	0%	0%	0%	0%	0%	0%	0%
	Type XIV	3	0%	0%	0%	0%	0%	0%	0%	0%	0%	0%	0%	0%	0%	0%	0%	0%

*Significantly up- or downregulated gene, log_2_(fold change) > 1 or < −1, respectively, adjusted p-value (FDR) < 0.05*.

*hpi, Hours post inoculation; L-lectin, L-type lectin; TLPK, thaumatin-like protein kinase; WAK, wall-associated kinase; RFK3-like, receptor-like kinase in flowers 3; PERK-like, proline-rich extensin-like receptor protein kinase; LRK10-like, leaf rust 10 disease-resistance locus receptor-like protein kinase; DUF26, domain of unknown function 26; CR4-like, crinkly4-like; LysM, lysin motif receptor-like kinase; C-lectin, C-type lectin*.

Domain of unknown function 26 (DUF26) and S-domain RLKs were among the largest families of identified RLKs in *P. americana* (Table 4). A substantial portion of these were significantly upregulated throughout all the time-points in Dusa®, following *P. cinnamomi* inoculation, and at 6 and 120 hpi in R0.12. Interestingly, more DUF26 and S-domain RLKs were significantly upregulated in R0.12 at 6 hpi when compared to Dusa®, while the opposite was observed at 120 hpi. Another large family of identified RLKs were wall-associated kinases (WAKs); of these, many were significantly upregulated at 6 hpi in both Dusa® and R0.12, while a smaller but notable proportion was downregulated. In Dusa®, the downregulated portion of WAKs decreased steadily over time, while the upregulated portion decreased at 12 and again at 24 hpi followed by an increase at 120 hpi. Interestingly, the vast majority of DE extensins were downregulated in both rootstocks.

Leucine-rich repeat RLKs were, by far, the largest family of identified RLKs in *P. americana* (Table 4). In contrast to the RLKs reported earlier, a large percentage LRR-RLKs, specifically of types I, VIII, and XII were significantly upregulated in Dusa® at the earliest time point but decreased thereafter. Similarly, a large proportion of these LRR-RLKs were upregulated at 6 hpi in R0.12, while less was observed at 120 hpi. The share of significantly upregulated type X and XIII LRR-RLKs was also high at 6 hpi but low at 120 hpi in both rootstocks. However, in Dusa®, the largest percentage of upregulated type X and XIII LRR-RLKs were seen at 24 hpi. Opposingly, the majority of DE types III, VII, and XI LRR-RLKs were downregulated at all the time-points in Dusa® and at 6 and 120 hpi in R0.12. The downregulated portion of DE types II and V LRR-RLKs remained consistent at all the time points in Dusa®, while the upregulated portion of these RLKs peaked at 12 hpi. In R0.12, a larger percentage of type II was downregulated at 6 and 120 hpi when compared to Dusa®. Lastly, the majority of DE types IV and VI LRR-RLKs were significantly downregulated at 120 hpi in both rootstocks. A small percentage of type VI was upregulated at 24 and 120 hpi in Dusa® and at 120 hpi in R0.12. It is worth noting, however, that at 12 and 24 hpi, there was almost no up- or downregulation of these or any other putative RLK coding genes in R0.12. If not for the 12 and 24 hpi time points, very few differences would be observed between R0.12 and Dusa®.

#### Apoplastic Proteases

Predicted proteases were extracted from the Mercator 3 annotation file. A total of 62 aspartic proteases, 77 cysteine proteases, 50 metalloproteases, and 229 serine proteases were recovered (Supplementary Table 30). Of the 229 serine proteases identified, 108 were subtilases (SBTs) and separated during subsequent analyses. This list was filtered to further exclude any proteases that did not show significant differential expression at any time or in any rootstock when compared to the appropriate mock-inoculated control. The subcellular localization of these DE proteases was predicted using an online web service, available at (http://embed.protein.properties). Twenty-five aspartic proteases were DE, of which 20 were predicted to localize to the extracellular space and two to the cell membrane. Interestingly, only 18 of the predicted cysteine proteases were DE, of which only three were expected to localize extra-cellularly. Altogether, 23 metalloproteases were DE; however, only five and two were predicted to localize to the extracellular space and the cell membrane, respectively. Forty-one non-SBT serine proteases were DE, of which 18 were expected in the extracellular space, and only one was predicted to localize to the cell membrane. In total, 37 SBTs were DE; surprisingly all the 37 were predicted to exist in the extracellular space. Only proteases predicted to localize n the extracellular space or in the cell membrane, henceforth collectively referred to as apoplastic proteases, were retained for further investigation.

**Figure 8:**
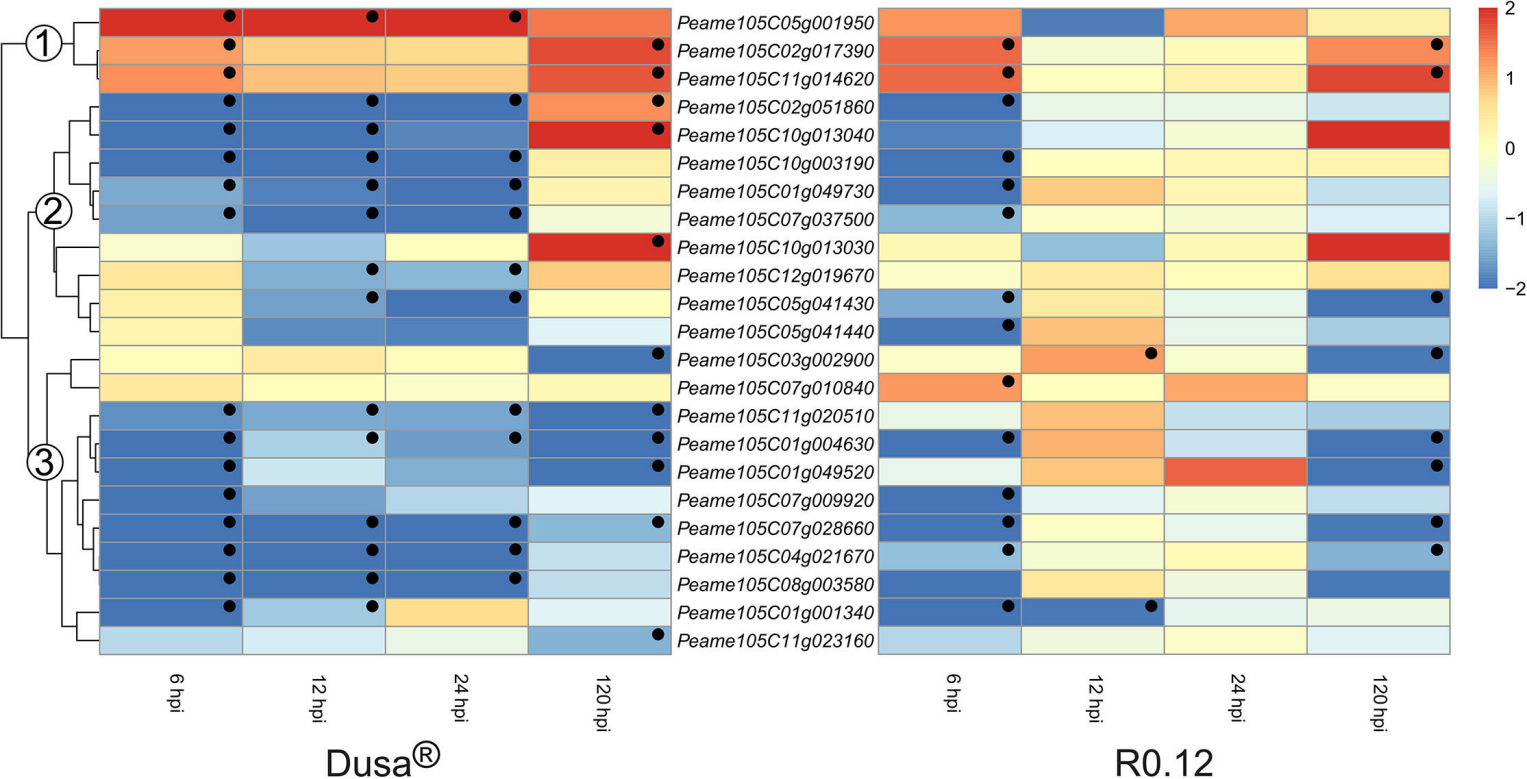
Heatmap and dendrogram illustrating the expression of genes encoding for apoplastic aspartic proteases in *Persea americana* following inoculation with *Phytophthora cinnamomi*. Differentially expressed genes (DEGs) encoding for apoplastic aspartic proteases from both partially resistant (Dusa®) and susceptible (R0.12) *P. americana* rootstocks are indicated. Differential expression at 6, 12, 24, and 120 h post inoculation (hpi), following inoculation with *P. cinnamomi*, was calculated using mock-inoculated (dH_2_O) sample libraries as the control. Significantly upregulated [log_2_(fold change) > 1, *p* = 0.05] genes are indicated by a red color, while significantly downregulated [log_2_(fold change) < −1, *p* = 0.05] genes are indicated in blue, the intensity of which represents scale. Significant DEGs are indicated with a black dot. The expression of aspartic protease coding genes was broadly clustered into three groups based on the dendrogram from Dusa® and were indicated numerically on the dendrogram. Only aspartic proteases that were differentially expressed in one or more samples and were predicted to localize to the cell membrane or extracellular space are shown.

Only a small number of genes encoding for apoplastic aspartic proteases were significantly upregulated in either rootstock at any given time (Figure 8). Three groups were assigned based on the clustering of genes on the dendrogram obtained from Dusa®. In group 1, genes were generally upregulated following *P. cinnamomi* challenge. One, *Peame105C05g001950*, was significantly upregulated at the three earliest time points in Dusa® when compared to the mock-inoculated control. However, in R0.12, this gene was not upregulated at any time-point but instead tended toward downregulation, although not significant, at 12 hpi. The remaining two were upregulated at 6 and 120 hpi in both Dusa® and R0.12. Most of the genes in group 2 were downregulated at 6-, 12, and 24 hpi in Dusa® and 6 hpi in R0.12. Three of these were significantly upregulated at 120 hpi in Dusa®, while only one reached significance in R0.12. Genes that fall in group 3 were generally downregulated at most time points in Dusa® and at 6 and 120 hpi in R0.12. Interestingly, two of these, *Peame105C07g010840* and *Peame105C03g002900*, were significantly upregulated in R0.12 at 6 and 12 hpi, respectively.

**Figure 9:**
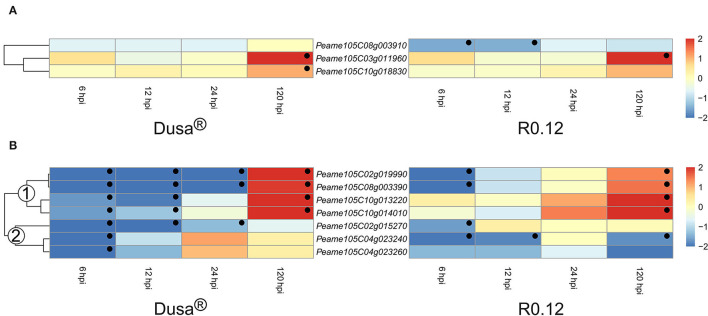
Heatmap and dendrogram illustrating the expression of genes encoding for apoplastic cysteine proteases and metalloproteases in *Persea americana* following inoculation with *Phytophthora cinnamomi*. Differentially expressed genes (DEGs) encoding for **(A)** apoplastic cysteine proteases and **(B)** apoplastic metalloproteases from both partially resistant (Dusa®) and susceptible (R0.12) *P. americana* rootstocks are indicated. Differential expression at 6, 12, 24, and 120 h post inoculation (hpi), following inoculation with *P. cinnamomi*, was calculated using mock-inoculated (dH_2_O) sample libraries as the control. Significantly upregulated [log_2_(fold change) > 1, *p* = 0.05] genes are indicated by a red color, while significantly downregulated [log_2_(fold change) < −1, *p* = 0.05] genes are indicated in blue, the intensity of which represents scale. Significant DEGs are indicated with a black dot. The expression of metalloprotease coding genes was broadly clustered into two groups based on the dendrogram from Dusa® and were indicated numerically on the dendrogram. Only cysteine proteases and metalloproteases that were differentially expressed in one or more samples and were predicted to localize to the cell membrane or extracellular space are shown.

Cysteine proteases and metalloproteases represented the two smallest groups of DE apoplastic proteases identified in this study. Of the cysteine proteases described, two were significantly upregulated at 120 hpi in Dusa®, while only one reached significance in R0.12 (Figure 9A). Intriguingly, *Peame105C08g003910* was significantly downregulated at 6 and 12 hpi in R0.12 but not in Dusa®. The heatmap describing the metalloproteases was divided into two groups based on the dendrogram obtained from Dusa® (Figure 9B). Metalloproteases in the first group contained four genes that were downregulated at 6 and 12 hpi in Dusa®, two of which were also downregulated at 24 hpi. The same two were downregulated in R0.12 but only at 6 hpi, while the remaining two were not DE at 6, 12, or 24 hpi. Conversely, all four were significantly upregulated in both Dusa® and R0.12 at 120 hpi. The second group of metalloproteases was mostly downregulated at various early time points in Dusa® and R0.12; one of these was also downregulated at 120 hpi in R0.12. Based on the avocado genome annotation, all except one DE apoplastic metalloproteinase, *Peame105C02g015270*, were predicted to encode for matrix metalloproteases (MMPs).

**Figure 10:**
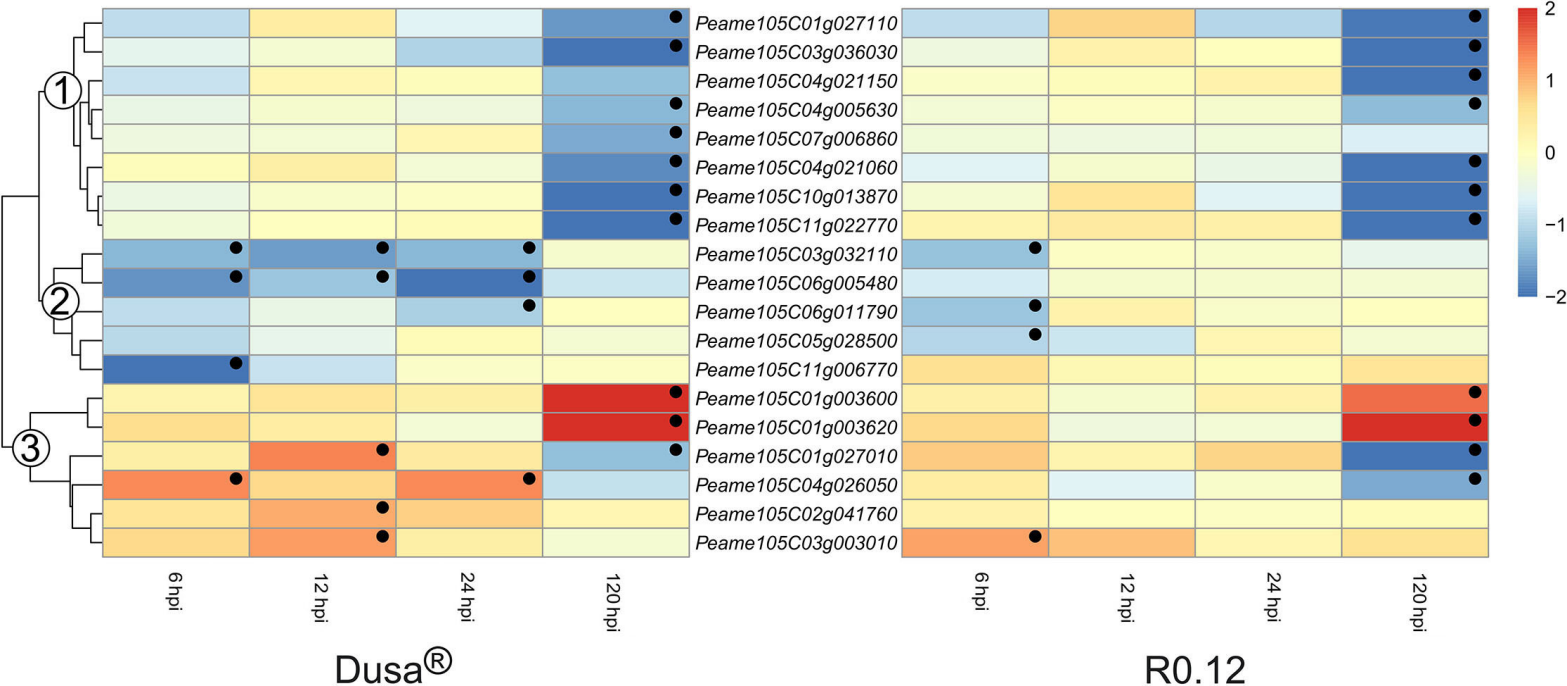
Heatmap and dendrogram illustrating the expression of genes encoding for apoplastic serine proteases in *Persea americana* following inoculation with *Phytophthora cinnamomi*. Differentially expressed genes (DEGs) encoding for apoplastic serine proteases from both partially resistant (Dusa®) and susceptible (R0.12) *P. americana* rootstocks are indicated. Differential expression at 6, 12, 24, and 120 h post inoculation (hpi), following inoculation with *P. cinnamomi*, was calculated using mock-inoculated (dH_2_O) sample libraries as the control. Significantly upregulated [log_2_(fold change) > 1, *p* = 0.05] genes are indicated by a red color, while significantly downregulated [log_2_(fold change) < −1, *p* = 0.05] genes are indicated in blue, the intensity of which represents scale. Significant DEGs are indicated with a black dot. The expression of serine protease coding genes was broadly clustered into three groups based on the dendrogram from Dusa® and were indicated numerically on the dendrogram. Only serine proteases that were differentially expressed in one or more samples and were predicted to localize to the cell membrane or extracellular space are shown.

The heatmaps depicting the expression of genes encoding for non-SBT serine proteases could be divided into three groups based on the dendrogram from Dusa® (Figure 10). The avocado genome annotation classified all but one, *Peame105C06g005480*, as encoding for serine carboxypeptidase-like proteins (SCLPs). Genes in the first group were not DE at 6, 12, or 24 hpi in either Dusa® or R0.12. However, all except one from each rootstock were significantly downregulated at 120 hpi. In comparison, genes from group 2 were significantly downregulated during various early time points in Dusa® and at 6 hpi in R0.12. However, neither rootstock displayed up- or downregulation for any group 2 apoplastic serine proteases at 120 hpi. For group 3, there were two genes that were significantly upregulated at 120 hpi in both rootstocks but none at any other time point. Three of the remaining group 3 serine protease coding genes were significantly upregulated in Dusa® at 12 hpi, one of which was significantly downregulated at 120 hpi. Only one of these, *Peame105C03g003010*, was significantly upregulated at an early time-point in R0.12. The last group 3 serine protease, *Peame105C04g026050*, was upregulated at 6 and 24 hpi in Dusa® while only being downregulated in R0.12 at 120 hpi. Although the same gene was inclined toward downregulation in Dusa® at 120 hpi, it did not reach significance.

**Figure 11:**
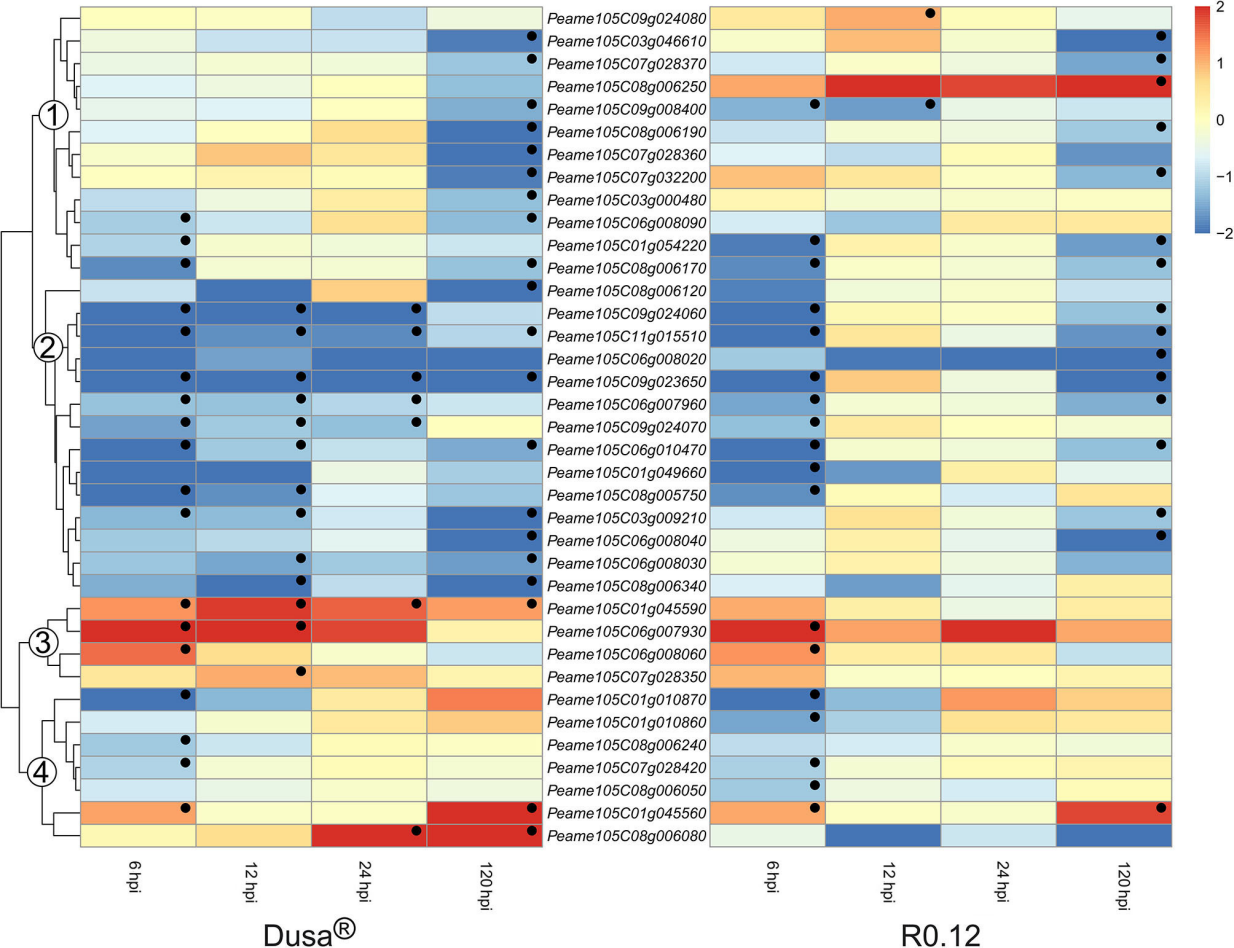
Heatmap and dendrogram illustrating the expression of genes encoding for apoplastic subtilases (SBTs) in *Persea americana* following inoculation with *Phytophthora cinnamomi*. Differentially expressed genes (DEGs) encoding for apoplastic SBTs from both partially resistant (Dusa®) and susceptible (R0.12) *P. americana* rootstocks are indicated. Differential expression at 6, 12, 24, and 120 h post inoculation (hpi), following inoculation with *P. cinnamomi*, was calculated using mock-inoculated (dH_2_O) sample libraries as the control. Significantly upregulated [log_2_(fold change) > 1, *p* = 0.05] genes are indicated by a red color, while significantly downregulated [log_2_(fold change) < −1, *p* = 0.05] genes are indicated in blue, the intensity of which represents scale. Significant DEGs are indicated with a black dot. The expression of SBT coding genes were broadly clustered into four groups based on the dendrogram from Dusa® and were indicated numerically on the dendrogram. Only SBTs that were differentially expressed in one or more samples and were predicted to localize to the cell membrane or extracellular space are shown.

Surprisingly, all significant DE SBTs were predicted to exist in the extracellular space; as such, they constitute the largest group of apoplastic proteases identified in *P. americana*. The expression of SBT coding genes could be broadly clustered into four groups based on the dendrogram from Dusa® (Figure 11). The genes found in group 1 from Dusa® were not DE at 6, 12, or 24 hpi; however, the majority was significantly downregulated at 120 hpi. In R0.12, the expression of genes in group 1 was largely comparable to Dusa®, except for three. The first, *Peame105C009g008400*, was significantly downregulated at 6 and 12 hpi; the second, *Peame105C09g024080*, was significantly upregulated at 12 hpi; the third, *Peame105C08g006250*, tended toward upregulation at 6, 12, and 24 hpi, achieving significance at 120 hpi. The second group of SBTs was generally downregulated at most time-points in Dusa® and at 6 and 120 hpi in R0.12. In contrast, group 3 contained genes that were significantly upregulated in Dusa® at several early time-points, especially 6 hpi. In R0.12, only two of these genes were significantly upregulated and only at 6 hpi. *Peame105C01g045590* was particularly interesting, as it was significantly upregulated in Dusa® at all the time-points; however, in R0.12 it was not DE at any. Based on Blastp, the predicted protein coding sequence for *Peame105C01g045590* and *Peame105C01g045560* closely resembled that of SBT3.3 in *Cinnamomum micranthum*. Lastly, group 4 consisted primarily of genes that were downregulated at 6 hpi in either Dusa® or R0.12 but not at any of the subsequent time points. Group 4 also contained one gene that was upregulated at 6 and 120 hpi in both rootstocks. Interestingly, the last member of group 4, *Peam105C08g006080*, was upregulated at 24 and 120 hpi in Dusa® but tended toward downregulation in R0.12. Here, the Blastp analysis revealed that the predicted protein sequence of *Peam105C08g006080* showed high similarity to SBT3 like proteins in *C. micranthum*.

## Discussion

The molecular basis of *P. cinnamomi* resistance in avocado has been the topic of a recent discussion (van den Berg et al., 2021). This review provided an up-to-date justification and classification of avocado rootstocks as resistant, partially resistant, tolerant, or susceptible to *P. cinnamomi*. The current study attempted to contextualize the basis of partial resistance by temporal transcriptomic analyses of representatively susceptible (R0.12) and partially resistant (Dusa®) avocado rootstocks inoculated with *P. cinnamomi* (Engelbrecht and van den Berg, 2013; van den Berg et al., 2018b). Using dual RNA sequencing technology, this study provides a holistic overview of both avocado and *P. cinnamomi* gene expression over time, during their interaction. Notably, sample libraries were collected for the characterization of gene expression at both early, 6, 12, and 24 hpi, and late, 120 hpi, responses. As expected, the relative abundance of transcripts that mapped back to the *P. cinnamomi* genome increased substantially between the early and late sample libraries in both rootstocks, indicating host colonization and spread of *P. cinnamomi*. Contrastingly, the number of avocado transcripts that mapped back to the *P. americana* genome decreased substantially at the late time points. This last observation was somewhat expected, given that root necrosis is often observed following *P. cinnamomi* inoculation of avocado rootstocks at later time points (Engelbrecht and van den Berg, 2013; Engelbrecht et al., 2013). Supportively, a previous study provided evidence that *P. cinnamomi* switches to a necrotrophic life stage at around 24 hpi in the rootstock Dusa®(van den Berg et al., 2018b).

In this study, the initial analyses revealed overwhelmingly significant differences in the number of avocado DEGs between rootstocks, specifically at 12 and 24 hpi. At these time points, the number of DEGs in the susceptible rootstock paled in comparison to DEGs in the partially resistant rootstock. In fact, in the susceptible rootstock, slightly more than 800 DEGs were identified when combining both time points, while over 13,000 were found in Dusa®. In contrast, at 6 and 120 hpi, both rootstocks displayed a similar number of up- or downregulated genes. The PCAs and hierarchical clustering of avocado DEGs further supported the variation in the transcriptomes of R0.12 and Dusa® at 12 and 24 hpi. In fact, the R0.12 12 and 24 hpi sample libraries were indistinguishable from the mock-inoculated sample libraries following PCA, while a clear difference was seen among mock, early, and late responses in Dusa®. Together, these observations provide the first indication of major transcriptomic differences between Dusa® and R0.12, specifically at 12 and 24 hpi, following inoculation with *P. cinnamomi*.

Following temporal cluster analyses of avocado DEGs, GO enrichment confirmed the activation of pathways related to defense responses in the gene sets from clusters that were upregulated at 6 hpi in both Dusa® and R0.12. Surprisingly, however, in R0.12, the expression of these genes seemed to revert to near mock-inoculated levels at 12 and 24 hpi. Instead, the cluster analyses and subsequent GO enrichment revealed that R0.12 was activating pathways related to growth and development at 24 hpi. In contrast, similar pathways were identified following the GO enrichment of the cluster representing genes that were downregulated at all post-inoculation time points in Dusa®. Instead, in Dusa®, the activation of pathways related to defense continued at 12 and 24 hpi. Clusters representing gene sets that were upregulated at 120 hpi in both Dusa® and R0.12 gave rise to similarly enriched GO terms. Here, the pathways related to phosphorylation were the most significantly enriched in both rootstocks. Notably, phosphorylation is known to play an essential role in plant immune responses, constituting a requisite part of PRR-mediated defense response signaling (Park et al., 2012). Otherwise, common defense-related terms such as response to chitin, oxidative stress, and wounding, were also enriched in these clusters. Together, this evidence points toward a high likelihood that most of the variation in resistance between R0.12 and Dusa® lies in the early response to *P. cinnamomi* challenge.

### P. cinnamomi

Interestingly, the increased expression of *P. cinnamomi* elicitin homologs, a class of effectors known for inducing HR (Derevnina et al., 2016), occurred far earlier in R0.12 than in Dusa® (Table 2). Similarly, the *P. cinnamomi* homologs of the phytotoxic *P.cactorum*-Fragaria (PcF) protein and xyloglucan-specific endo-beta-1,4-glucanase 1 (XEG1) were expressed at 24 hpi in R0.12 but not in Dusa®. Notably, XEGs are an important group of cell wall-degrading enzymes (CWDEs); *Phytophthora sojae* XEG1 (PsXEG1) was shown to be an important determinant of virulence in infected soybean (Ma et al., 2015, 2017; Xia et al., 2020). Meanwhile, PcF application was revealed to cause necrosis in both tomato and strawberry (Orsomando et al., 2001). Together, these observations suggest that the necrotrophic life stage of *P. cinnamomi* may initiate earlier in R0.12 following inoculation when compared to Dusa®. This hypothesis should constitute the focus of a separate study given the possible implications for the industry.

The effector proteins used by *P. cinnamomi* showed further variance when comparing Dusa® and R0.12, exemplified by the upregulation of putative RxLR effector coding genes. RxLRs are an important class of cytoplasmic effectors in *Phytophthora* species and the subject of extensive study (Win et al., 2007; Jiang et al., 2008; McGowan and Fitzpatrick, 2017; Reitmann et al., 2017; Hardham and Blackman, 2018; Engelbrecht et al., 2021; Joubert et al., 2021). Although a large proportion of the upregulated RxLR coding genes in either Dusa® or R0.12 were shared between the rootstocks, each was host to a unique compliment. Interestingly, in R0.12, the largest compliment of upregulated RxLR coding genes was found at 24 hpi. Meanwhile, the number and diversity of upregulated RxLR coding genes in Dusa® increased steadily as the infection progressed. Importantly, *Phytophthora* RxLRs are known to play a fundamental role in the evasion of host defense signaling and immune responses (Kelley et al., 2010; Vetukuri et al., 2017; Dalio et al., 2018; Naveed et al., 2019). Thus, it is reasonable to assume that the unique compliment of upregulated RxLR coding genes in R0.12 is at least partly responsible for the inappropriate response seen in this rootstock at 12 and 24 hpi. However, the upregulated RxLR coding genes in Dusa® should not be overlooked given that this rootstock is not fully resistant to *P. cinnamomi*. For more details on the expression of the putative *P. cinnamomi* RxLR effector coding gene repertoire, using some of the data from this study, readers are referred to the study performed by Joubert et al. (2021).

Although fewer in number, several other important groups of effectors were upregulated in both Dusa® and R0.12, and showed a varied expression between the rootstocks over time. The first of these, the NLP effectors, represent a large family of microbial effectors that contribute significantly to virulence and host defense response elicitation (Pemberton and Salmond, 2004; Seidl and van den Ackerveken, 2019). Intriguingly, only half of the upregulated putative *NLP*s were shared between Dusa® and R0.12. Additionally, more upregulated NLP coding genes were identified in R0.12 at 6 and 24 hpi than in Dusa®, while more were identified in Dusa® at 120 hpi. Together, these observations would suggest that the NLPs likely contribute to differences in the observed virulence of *P. cinnamomi* on Dusa® and R0.12.

Entirely unique compliments of upregulated CRN effector coding genes were seen when comparing *P. cinnamomi* gene expression in Dusa® and R0.12 (Table 2). CRNs are a large, functionally diverse family of modular cytoplasmic effectors that have been described in a wide array of plant-pathogenic oomycetes (Tyler et al., 2006; Kamoun, 2007; Haas et al., 2009; Schornack et al., 2010; Stam et al., 2013; McGowan and Fitzpatrick, 2020). These effectors were first identified in *Phytophthora infestans* for their ability to induce host leaf-crinkling and necrosis as well as induce defense responses (Torto et al., 2003). Since then, CRNs have also been implicated in the suppression of host defense responses (Chen et al., 2013; Mafurah et al., 2015; Xiang et al., 2021). It is worth noting that 45 *CRN*s were previously identified from the *P. cinnamomi* genome (Engelbrecht et al., 2021). However, only five upregulated *CRN*s were identified in the current study using the PHI database. Given the apparent importance of this group of effectors, it seems unlikely that the current study accurately described the full extent of *CRN* expression in *P. cinnamomi*. Nonetheless, differences were still seen in the number and temporal association of expressed *CRN*s, warranting further fine-grained analyses and characterization.

Interestingly, substantially more upreguated *GIP*s were observed in Dusa® at 120 hpi than in R0.12. In studied *Phytophthora* spp., GIPs bind to and inhibit plant secreted endo-β-1,3-glucanases (EGases; Damasceno et al., 2008). Notably, EGases act on an abundant component of *Phytophthora* cell walls, β-1,3 glucan, directly damaging the pathogen while releasing PAMPs known to elicit host defense responses (Erwin et al., 1983; van Loon et al., 2006). Markedly, β-1,3-glucanase has been linked to increased *P. cinnamomi* resistance in the partially resistant rootstock R0.06 (van den Berg et al., 2018a). Thus, it is possible that the EGases produced by Dusa® and not R0.12 might have led to the consequent upregulation of *P. cinnamomi* GIPs at later time points, to directly counteract cell wall damage and the release of related PAMPs.

Similarly, more upregulated EPI effector coding genes were seen at both 24 and 120 hpi in *P. cinnamomi-*inoculated Dusa® than in R0.12. Notably, EPI effectors are known to inhibit plant-derived extracellular proteases. For example, the *P. infestans* effectors EPI1 and EPI10 interact with and inhibit the apoplastic serine protease P69B (Tian et al., 2004, 2005). Similarly, the homolog from *Phytophthora palmivora*, EPI10, inhibits the extracellular subtilase HbSPA from *Hevea brasiliensis* (Ekchaweng et al., 2017). Inhibition of plant proteases seems to be an advantageous strategy that has developed in diverse plant pathogenic species such as *Cladosporium fulvum*, which secretes the effector Avr2 (Rooney et al., 2005). This effector has been shown to inhibit several apoplastic cysteine proteases in tomato, including Required for *Cladosporium* resistance 3 (Rcr3) and *Phytophthora-*inhibited protease 1 (Rooney et al., 2005; Shabab et al., 2008; van Esse et al., 2008; Pip1). Similarly, the *P. infestans* cystatin-like EPIs EPIC1 and EPIC2B are also known to target apoplastic proteases, including Rcr3 and Pip1 (Tian et al., 2007; Song et al., 2009). It would be reasonable to assume, given the larger compliment of upregulated EPIs observed following the inoculation of Dusa®, that *P. cinnamomi* might be responding to a larger or more devastating set of secreted proteases from the partially resistant rootstock.

Finally, several *CBEL*s were upregulated in both Dusa® and R0.12, most prominently at 6 hpi. This is in keeping with the primary function of CBELs, which allow *Phytophthora* mycelium to adhere to cellulosic structures such as the cell wall of plants (Mateos et al., 1997; Gaulin et al., 2002). However, CBELs are also known to elicit a strong host defense response, acting like PAMPs (Gaulin et al., 2006). As such, there has been some interest in production of recombinant CBEL proteins for agricultural applications as activators of plant immune responses (Larroque et al., 2011). Thus, the CBELs identified here might be deemed novel targets for future research aimed at understanding the early determinants of *P. cinnamomi* virulence in avocado and increasing resistance to this devastating pathogen.

### P. americana

Given that PRRs constitute the starting point for inducible defense responses in plants, it is conceivable that the overwhelming lack of defense responses at 12 and 24 hpi in R0.12 might stem from inappropriate PRR signaling or activation. To this end and given the prevalence of GO terms related to phosphorylation in both Dusa® and R0.12, we sought to characterize differences in the expression of major classes of RLKs in avocado. When considering the representative percentages of up- or downregulated *RLK* families at 6 and 120 hpi in Dusa® and R0.12, very few substantiative differences were noted between the rootstocks. This fact limited the usefulness of a direct comparison between the rootstocks, given the broad approach used in this study. However, when considering only the DE of *RLK*s in Dusa®, some interesting patterns emerged. For instance, some *RLK*s, such as those encoding for extensins and LRR-RLKs belonging to types III, IV, VII, and XII protein families, were substantially downregulated across several or all time points. Although RLKs are often considered PRRs, it is important to note that many have diverse roles in a myriad of non-pathogen-related functions (Breiden and Simon, 2016). Thus, it is likely that a vast number of *RLK*s in these families might either be linked to growth and development or are otherwise disadvantageous during *P. cinnamomi* challenge. There were also *RLK*s that were mostly upregulated during the earlier time points, such as types I, VIII, X, XII, and XIII *LRR*-*RLK* gene families, while contrastingly, genes encoding for L-lectin, TLPK, RFK3-like, PERK-like, LRK10-like, and CR4-like RLK proteins were, by and large, upregulated at later time-points. Of note, RLKs have diversly described roles against a multitude of biotrophic, hemibiotrophic, and necrotrophic pathogens (Llorente et al., 2005; Veronese et al., 2005; Roux et al., 2011; Mosher et al., 2013; Zhang et al., 2013a,b). Thus, it is interesting to broadly posit roles for many of these *RLK* gene families in either early (biotrophic) or late (necrotrophic) responses to *P. cinnamomi* based on their temporal affinity. It might also be worthwhile to determine the roles of RLK coding genes, such as S-domain, DUF26, WAK-like, and types II, V, and VII LRR-RLKs, of which a large percentage were upregulated at all time-points following *P. cinnamomi* inoculation.

The advent of PTI is also often associated with increased callose deposition at the site of infection (Voigt, 2014). Interestingly, in the partially resistant avocado rootstock R0.06, challenged by *P. cinnamomi*, confocal microscopy noted rapid deposition of callose as early as 6 hpi near the site of infection (van den Berg et al., 2018a). In contrast, some scant callose deposition was only noted at 96 hpi in the susceptible rootstock R0.12. A similar observation has been made for Dusa®, in which *P. cinnamomi* inoculation leads to rapid accumulation of callose near the site of infection (unpublished study, personal communication). Surprisingly, DE callose synthase genes were not identified from transcriptomic data (van den Berg et al., 2018b). Nevertheless, it has been suggested that the regulation of callose biosynthesis might not occur primarily at the transcriptional level but rather at the translational level or by the involvement of proteases (Nakashima et al., 2003; Saheed et al., 2009). In support of this hypothesis, *Arabidopsis thaliana* plants overexpressing *Vitis quinquangularis* aspartic protease 13 (*VqAP13*) showed enhanced callose deposition at the site of *Pseudomonas syringae* pv. *tomato* (*Pst*) DC3000 inoculation when compared to wild-type plants (Guo et al., 2016). Similarly, overexpression of the *Oryza sativa* matrix metalloprotease OsMMP1 in tobacco led to increased callose and cellulose deposition (Das et al., 2018). Thus, it is entirely possible that enhanced callose deposition in some partially resistant avocado rootstocks may, to some extent, be regulated by proteases. In the present study, several proteases were significantly upregulated at 6 hpi in Dusa® but not in R0.12, including the aspartic protease *Peame105C05g001950*, the serine protease *Peame105C04g026050*, and the subtilase *Peame105C01g045590*. Given the early onset (6 hpi) of enhanced callose deposition in partially resistant rootstocks, these proteases may deserve further study in this regard along with the non-apoplastic proteases that were not looked at in this study. Nonetheless, elucidating the role of these and other avocado proteases in callose deposition as well as other aspects of PTI should constitute the topic of future investigations.

Otherwise, several subtilases in either rootstock were significantly upregulated at 6 hpi when compared to the mock-inoculated control plants, and while the susceptible rootstock R0.12 increased the expression of three subtilases at 6 hpi, four were upregulated in Dusa®. Several subtilases were also significantly upregulated at 12 and 24 hpi in Dusa® but not in R0.12. These increases occurred during the theoretical biotrophic phase of *P. cinnamomi*'s life cycle; as such it is tempting to posit that they might play a role in inducing ETI and subsequent HR. HR, characterized by rapid localized cell death at the initial site of pathogen infiltration, constitutes an effective strategy for defense against biotrophic pathogens and early interactions with hemibiotrophic pathogens (Balint-Kurti, 2019). In animals, caspases (cysteine-containing aspartate-specific proteases) play an important part in the control of PCD (Boatright and Salvesen, 2003). Interestingly, several proteases, specifically apoplastic subtilases, have been implicated in caspase-like activity in plants, promoting cell death during pathogenic challenge (Coffeen and Wolpert, 2004; Chichkova et al., 2010; Vartapetian et al., 2011; Fernández et al., 2012; Zimmermann et al., 2016). In tomato, the subtilase P69B was induced by both biotrophic and hemibiotrophic pathogens, as well as salicylic acid (SA) application (Fischer et al., 1989; Tornero et al., 1996; Jordá et al., 1999; Jord and Vera, 2000). In an elegant study, Zimmermann et al. (2016) showed that P69B promoted cell death in tomato, although the overexpression of P69B alone could not. Thus, control over PCD is an undoubtedly important aspect of defense responses, and apoplastic subtilases form an integral part of its control, although additional proteases also participate (Godson and van der Hoorn, 2021). Investigations aimed at determining whether these subtilases display caspase-like activity would be highly informative.

It was worth noting that two tomato MMPs, Sl2-MMP and Sl3-MMP, act antagonistically upstream of P69B, cleaving it to prevent cell death (Zimmermann et al., 2016). Interestingly, in the current study, all the identified apoplastic metalloproteases were downregulated in Dusa® at 6 hpi, while two in R0.12, *Peame105C10g014010* and *Peame105C10g013220*, were not. The same metalloproteases were then significantly upregulated in both rootstocks at 120 hpi. This pattern of expression would come as no surprise if one were to first presume, as was the case for Sl2-MMP and Sl3-MMP, that these metalloproteases suppress cell death. It, therefore likely that further investigations might reveal similar functions for the avocado metalloproteases described in this study, and that the initiation of HR in Dusa® is more strongly induced as a result.

Some extracellular proteases have been implicated in the regulation of systemic acquired resistance (SAR) and priming of host immune responses (Xia et al., 2004; Prasad et al., 2009; Ramírez et al., 2013; Breitenbach et al., 2014). Pertinently, extracellular aspartic proteases in both tobacco and tomato have been associated with degradation of PR proteins (Rodrigo et al., 1989, 1991). Thus, it was previously suggested that an extracellular aspartic protease may be responsible for the cleavage of PR-1b (Hou et al., 2018). The resulting C-terminal PR-1b fragment, CAP-Derived Peptide 1 (CAPE1), has been defined as a DAMP and linked to the activation of jasmonic acid (JA) and SA defense response pathways, as well as SAR activation (Chen et al., 2014). Additionally, the aspartic protease Constitutive Disease Resistance 1 (CDR1) has been implicated in inducing SAR both locally and systemically (Xia et al., 2004; Prasad et al., 2009), while others such as apoplastic, EDS1-dependent 1 (AED1) serve to limit SAR signaling (Breitenbach et al., 2014). In *A. thaliana*, the subtilase SBT3.3 was found to participate in chromatin remodeling of SA defense-related genes, activating them following pathogen challenge and priming future immune response (Ramírez et al., 2013). Meanwhile, the *V. quinquangularis* aspartic protease coding gene *VqAP13* has been positively associated with the SA defense response pathway (Guo et al., 2013, 2016). Thus, the differences seen between R0.12 and Dusa® regarding differential expression of aspartic proteases and subtilases cannot be overlooked. For instance, it is conceivable that inappropriate expression of an *AED1* homolog in R0.12 could lead to suppression of defense responses in favor of growth. In contrast, upregulation of avocado *AP13, CDR1*, or *SBT3.3* homolog in Dusa® might lead to increased SAR or priming of SA defense response genes systemically. Congruently, it was previously shown that the SA defense response is an important part of the early immune response in Dusa® challenged by *P. cinnamomi* (van den Berg et al., 2018b). Interestingly, the predicted protein coding sequences for *Peame105C01g045560* and *Peame105C01g045590* closely resemble SBT3.3 in *C. micranthum*. Similarly, the product of *Peame105C08g006080* showed high similarity to several SBT3-like proteins in *C. micranthum*. Based on the near opposite regulatory patterns seen for two of these genes, *Peame105C01g045590* and *Peame105C08g006080*, in Dusa® and R0.12, further investigation is warranted. It would be interesting to determine whether the overexpression of these genes has the potential to enhance disease resistance and prime immune responses in a model system such as *A. thaliana* challenged by the oomycete *Hyaloperonospora arabidopsidis* or bacterium *Pst* DC3000.

Besides being one of the most well-studied plant proteases, SBTs are most often secreted (Schaller et al., 2018). Quite notably, the predicted proteins of all DE avocado apoplastic *SBT*s were expected to localize to the extracellular space. Additionally, all but one DE avocado apoplastic serine protease coding gene, excluding *SBT*s, was predicted to code for serine carboxypeptidase-like proteins (SCLPs). Markedly, SCLPs are known to contain signal peptides and are quite often found in the apoplast (Fraser et al., 2005; Sueldo et al., 2014; Grosse-Holz et al., 2018). Similarly, MMPs from the M10 family in plants have all been described as containing signal peptides and are the only metalloproteases in plants that are classed as apoplastic (Godson and van der Hoorn, 2021). Therefore, it is interesting to note that all but one DE avocado apoplastic metalloprotease coding gene was predicted to belong to the M10 MMP family. Together these observations provide support for the accuracy of the web service used to predict the subcellular localization of the proteases in this study (Stärk et al., 2021) and the relevance of the identified DE avocado apoplastic proteases for future characterization studies.

## Conclusion

In the current study, the basis of *P. cinnamomi* partial resistance in avocado was explored by dual RNA sequencing of a susceptible (R0.12) and a partially resistant rootstock (Dusa®). The initial analyses revealed significant differences in the number of DEGs between the rootstocks, particularly at 12 and 24 hpi. The GO enrichment indicated that while Dusa® activated various defense responses during these times, R0.12 activated pathways related to growth and development. *P. cinnamomi* DEGs reflected this difference, indicating that the use of effector proteins may either be causing or responding to the lack of defense responses in R0.12 at 12 and 24 hpi. In either case, the lack of appropriate defense responses from R0.12 at these intermediate time points coincided with the expected window in which *P. cinnamomi* switches between its biotrophic and necrotrophic life stages. In an attempt to uncover the root cause of such strikingly different responses, we also described broadly the expression of several avocado genes encoding RLKs and apoplastic proteases. To the best of our knowledge, no literature currently describes two plants of the same species, differing in their susceptibility to a pathogen, with such overwhelmingly different responses. As such, understanding the root cause of such a sweeping change in gene expression could provide novel insights into the avocado-*P. cinnamomi* interaction and the basis of partial resistance, and should preface investigations going forward. It should be noted that this study only used a single mock-inoculated avocado control time point because of resource restraints. Additionally, the use of a single susceptible and partially resistant rootstock limited the potential to highlight additional defense response pathways. Future investigations should endeavor to include controls at each time point to strengthen statistical analyses and use several rootstocks varying in their susceptibility to *P. cinnamomi* to provide a more holistic view of avocado defense responses following inoculation. Finally, it should be noted that low read counts were obtained for *P. cinnamomi* at the earliest time points. This limitation and potential cause for high inter-sample variation should be noted when considering the conclusions made in this study on *P. cinnamomi*.

## Data Availability Statement

The raw data for all RNA-sequencing sample libraries used in this study are available from the Sequence Read Archive of NCBI Genbank under accession number PRJNA675400.

## Author Contributions

NB, JE, and RB: conceptualized and reviewed the manuscript. JE: performed all the wet work. RB: performed all the data analyses and drafted the manuscript. All authors contributed to and approved the final version of the manuscript.

## Funding

This study was supported by the University of Pretoria and the Forestry and Agricultural Biotechnology Institute (FABI). Funding was provided by the Hans Merensky Foundation.

## Conflict of Interest

The authors declare that the research was conducted in the absence of any commercial or financial relationships that could be construed as a potential conflict of interest.

## Publisher's Note

All claims expressed in this article are solely those of the authors and do not necessarily represent those of their affiliated organizations, or those of the publisher, the editors and the reviewers. Any product that may be evaluated in this article, or claim that may be made by its manufacturer, is not guaranteed or endorsed by the publisher.

## Supplementary Material

The Suppl ementary Material for this article can be found online at: https://www.frontiersin.org/articles/10.3389/fpls.2022.928176/full#supplementary-material
